# Dynamic control of chirality in phosphine ligands for enantioselective catalysis

**DOI:** 10.1038/ncomms7652

**Published:** 2015-03-25

**Authors:** Depeng Zhao, Thomas M. Neubauer, Ben L. Feringa

**Affiliations:** 1 Centre for System Chemistry, Stratingh Institute for Chemistry, Faculty of Mathematics and Natural Sciences, University of Groningen, Nijenborgh 4, 9747AG Groningen, The Netherlands

## Abstract

Chirality plays a fundamental role in biology and chemistry and the precise control of chirality in a catalytic conversion is a key to modern synthesis most prominently seen in the production of pharmaceuticals. In enantioselective metal-based catalysis, access to each product enantiomer is commonly achieved through ligand design with chiral bisphosphines being widely applied as privileged ligands. Switchable phosphine ligands, in which chirality is modulated through an external trigger signal, might offer attractive possibilities to change enantioselectivity in a catalytic process in a non-invasive manner avoiding renewed ligand synthesis. Here we demonstrate that a photoswitchable chiral bisphosphine based on a unidirectional light-driven molecular motor, can be used to invert the stereoselectivity of a palladium-catalysed asymmetric transformation. It is shown that light-induced changes in geometry and helicity of the switchable ligand enable excellent selectivity towards the racemic or individual enantiomers of the product in a Pd-catalysed desymmetrization reaction.

## Abstract (web summary)

Typically in asymmetric catalysis each product enantiomer is produced using a different enantiomer of catalyst. Here, the authors show a photoswitchable bisphosphine ligand, capable of altering the stereoselectivity of a palladium catalysed process and producing either enantiomer of product.

## Graphical abstract

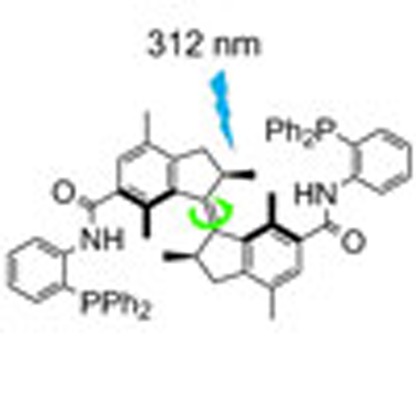

Responsive catalytic systems in which reactivity or selectivity can be modulated via external non-invasive signals are highly promising to enable tuning of catalytic function, allow spatio-temporal control in chemical transformations and ultimately arrive at multitasking catalysts. In recent years, the development of stimuli- responsive catalysts has attractive considerable attention^1^,^2^,^3^ and important efforts are related to on–off switching of catalytic activity^4^,^5^. Remarkable reversal of enantioselectivity in asymmetric catalysis has been achieved using solvent responsive helical polymers^6^, light-triggered organocatalysts^7^,^8^ and redox sensitive metal complexes^9^. These findings offer intriguing opportunities towards the design of broadly applicable responsive catalysts realizing that chiral transition metal catalysis centres among the most powerful and widely used methodologies to access enantiomerically pure compounds^10^. The asymmetric transformations rely on the well-defined chirality provided by the metal-bound ligands with a prominent role for chiral bisphosphines that encompass huge structural diversity^11^. A major challenge in asymmetric catalysis is to use a single enantiomer of a ligand or catalyst to obtain each enantiomer of a chiral product on demand^12^,^13^. Reported methodologies in which switching of enantioselectivity is observed are usually serendipitous or restricted to specific modifications, which include changing metals, solvents, temperature or other reaction parameters^14^,^15^,^16^,^17^,^18^,^19^,^20^. To achieve dual stereocontrol, we envision a rational and more general approach by designing chiral phosphine ligands that can exist in two (pseudo-)enantiomeric forms, which can be interconverted by light.

Although it is difficult to photochemically switch the chirality of conventional chiral ligands, artificial light-driven molecular motors provide a unique platform to achieve this goal^21^,^22^,^23^. Unidirectional rotary molecular motors based on overcrowded alkenes are intrinsically multistage chiral switches as we have recently shown in the design of three-stage organocatalysts^7^,^8^.

Herein, we demonstrate that a chiral bisphosphine with an intrinsic molecular motor core structure can be used as a multistage chiral switchable ligand for Pd catalysis. The stereoselectivity in a catalytic desymmetrization reaction involving an allylic substitution can be readily modulated by switching the helicity of the bisphosphine ligand on irradiation. This allows access to both enantiomers as well as the racemate of a functionalized cyclopentene using a single chiral ligand and these findings bring responsive chiral catalysts into the vast domain of phosphine-based transition metal catalysis^10^,^24^.

## Results

### Design and synthesis

**Figure 1:**
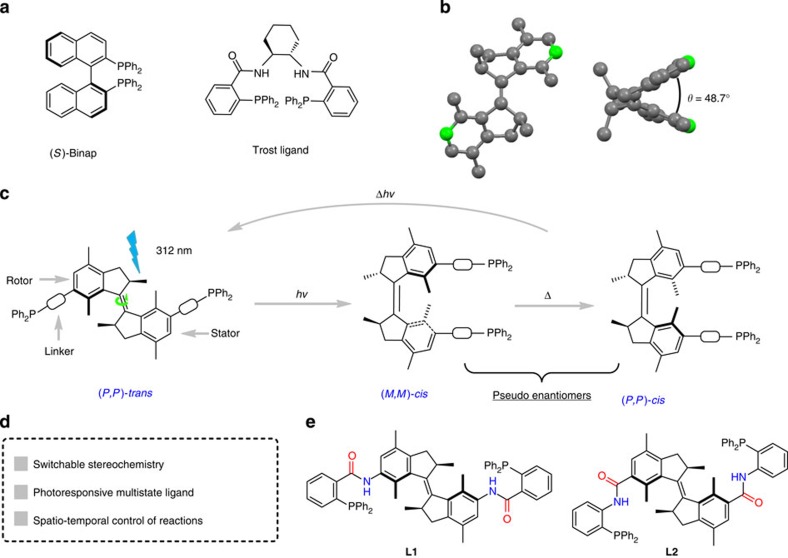
Chiral photoresponsive phosphine ligands. (**a**) Traditional *C*_2_-symmetric privileged ligands: (*S*)-Binap and Trost ligand. (**b**) Crystal structure of the parent molecular motor^25^; left: (*S*,*S*)-(*M*,*M*)*-trans* isomer, right: (*R*,*R*)-(*P,P*)*-cis* isomer. (**c**) Novel three-state photoresponsive phosphine ligand based on unidirectional molecular motor. The (*P*,*P*)-*trans* ligand based system might actually represent several catalytic complexes in the multi-state system, that is, oligomeric/polymeric P-Pd-ligand-Pd-ligand-Pd species due to phosphine ligand coordination in a monodentate manner to the metal. (**d**) Key features of novel chiral photoresponsive phosphine ligands. (**e**) Two potential photoresponsive phosphine ligands **L1** and **L2** incorporating amide linkers. *R* and *S* are used to define the chirality of the stereogenic centres; *P* (plus) and *M* (minus) are used to define the helicity of right and left-handed helix, respectively.

As a starting point for ligand design, we noted the advantages of *C*_2_-symmetric structures in many privileged bisphosphine ligands (Fig. 1a) and reasoned that a first generation molecular motor with two pending phosphine moieties using an appropriate linker might provide an optimal choice to achieve dynamic control over geometry and helicity of such bidentate chiral ligands (Fig. 1b,c). The structural design for the *C*_2_-symmetric bisphosphine ligands **L1** and **L2** with unique dynamic properties is shown in Fig. 1e.

The light-driven molecular motor unit is expected to undergo a unidirectional four-stage rotation in a 360° rotary cycle reaching (*P,P*)*-trans*, (*M,M*)*-cis*, (*P*,*P*)*-cis* and (*M*,*M*)*-trans* states, inducing both large geometrical and helical changes^21^,^22^. This allows both the spatial distance of the phosphine moieties and the chirality to be precisely controlled in each step of the cycle. We anticipated that in the *trans*-isomers the two phosphine groups are far apart from each other and unable to achieve intramolecular metal coordination, whereas in the *cis-*isomers they can effectively cooperate to form a chiral bisphosphine–metal complex (Fig. 1c). As the (*P,P*)*-cis* ligand–metal complex and (*M,M*)*-cis* ligand–metal complex are pseudo enantiomers, it can be expected that chiral products with opposite absolute configuration are obtained when these isomers are used in a catalytic asymmetric transformation. A crucial design feature is the nature of the linker moiety between motor and phosphine: (i) The linker should not contain additional chiral moieties to avoid match-mismatch effects for the *cis-*isomers and be relatively short so that the chiral orientation of the phosphines reflects the chirality of the motor; (ii) Small dihedral angle of the two phenyl rings in the *cis*-states based on the previous published crystal structure of a related first generation motor (Fig. 1b, 48.7° for (*P,P*)*-cis* isomer)^25^. The dihedral angle is closely related to the bite angle of the ligand–metal complex (P–metal–P angle), which is known to influence the catalytic behaviour and stereoselectivity in many asymmetric transformations^26^,^27^. In view of these considerations, amide linkers were chosen taking inspiration from Trost-ligands^28^, as these chiral ligands feature bisphosphines, amide linkers and in several cases show good tolerance for the chiral core units and are effective in numerous asymmetric transformations^29^. **L1** incorporates the amide linker in the same bond order as for the Trost ligand as shown in Fig. 1a, whereas **L2** adopts the reversed amide connection (Fig. 1e).

**Figure 2:**
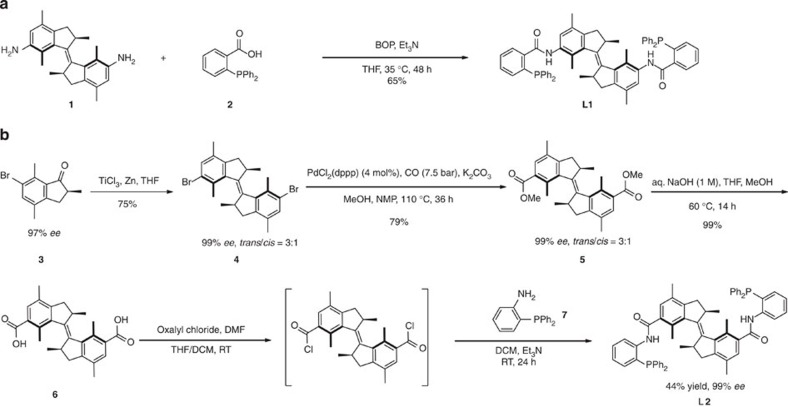
Procedure for the synthesis of ligands L1 and L2. (**a**) Procedure for synthesis of bisphosphine ligand (*P*,*P*)*-trans*-**L1**. (**b**) Asymmetric synthesis of bisphosphine ligand (*R*,*R*)*-*(*P*,*P*)*-trans*-**L2**. (For details, see Supplementary Methods).

The synthesis of molecular motor **L1** involved coupling of diamine **1** (ref. 8) with 2-(diphenylphosphino) benzoic acid **2** in the presence of BOP reagent ((Benzotriazol-1-yloxy)tris(dimethylamino)phosphonium hexafluorophosphate) and triethylamine (TEA) to afford bisphosphine **L1** in 65% yield (Fig. 2a). The asymmetric synthesis of bisphosphine ligand (*R*,*R*)-(*P*,*P*)-*trans*-**L2** started from chiral ketone **3**, which was prepared according to our recently reported gold-catalysed enantioselective protonation of the corresponding silyl enol ether in 97% *ee* (refs 30, 31). McMurry coupling using titanium trichloride and zinc provided the chiral dibromo motor **4** as a *trans*/*cis* mixture in 75% yield (*trans*/*cis*=3/1; 99% *ee* for both isomers). Subsequent palladium-catalysed carbonylation of dibromo motor **4** afforded diester **5** in 79% yield (*trans*/*cis*=3/1; isolated yield for the separated isomers: 59% for (*P*,*P*)-*trans*-**5** and 20% for (*P*,*P*)-*cis*-**5**). Hydrolysis of (*P*,*P*)-*trans* diester **5** with aq. NaOH gave diacid **6** in nearly quantitative yield as a single enantiomer. Treatment of the diacid **6** with oxalyl chloride in a mixture of THF and CH_2_Cl_2_ with catalytic amount of DMF gave the acid chloride intermediate, which was directly used in the next condensation step with (2-aminophenyl)diphenylphosphane **7** to furnish the final product **L2** in 44% yield over two steps. Molecular motor-based bisphosphines **L1** and **L2** were characterized by ^1^H, ^13^C and ^31^P NMR spectroscopy and high-resolution mass spectrometry (see Supplementary Figs 3–16).

### Photochemical and thermal isomerization

**Figure 3:**
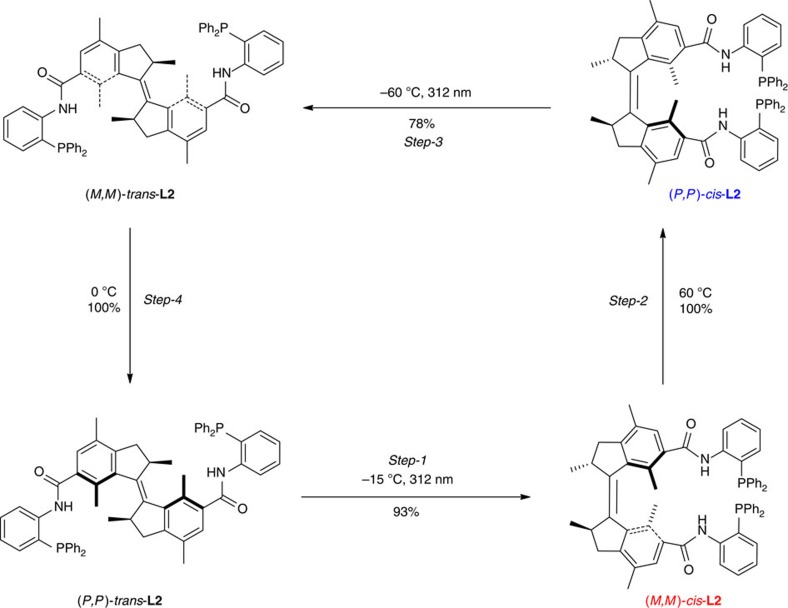
Schematic representation of unidirectional four-step rotary cycle of molecular motor L2. Step-1: photoisomerization of (*P*,*P*)-*trans*-**L2** resulted in (*M*,*M*)-*cis*-**L2** in which the two phosphine groups are brought into proximity to one another. Step-2: thermal helix inversion of (*M*,*M*)-*cis*-**L2** provides (*P*,*P*)-*cis*-**L2** in which the helicity changed from *M* to *P* while the two phosphine groups remain spatially in proximity. Step-3 and step-4: Photoisomerization of (*P*,*P*)-*cis*-**L2** and subsequent thermal helix inversion of (*M*,*M*)-*trans*-**L2** completes the full cycle.

**Figure 4:**
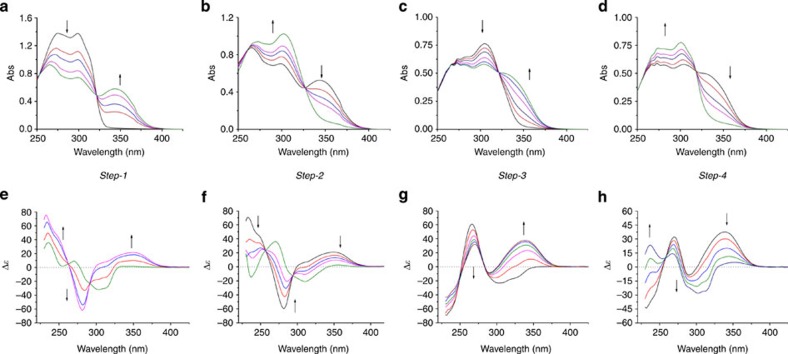
Ultraviolet–visible and CD spectral changes of L2 in each isomerization step. (**a**) Ultraviolet–visible spectral changes during step-1 on irradiation at 312 nm in THF at −15 °C. (**b**) Ultraviolet–visible spectral changes during step-2 on heating in THF at 60 °C. (**c**) Ultraviolet–visible spectral changes during step-3 on irradiation at 312 nm in THF at −60 °C. (**d**) Ultraviolet–visible spectral changes during step-4 (*t*_½_=11 min at 0 °C) in THF at 0 °C. Isosbestic points of step-1 to step-4: 321, 327, 322 and 318 nm. (**e**–**h**) The corresponding CD spectral changes during each of the four isomerization steps, respectively.

**Figure 5:**
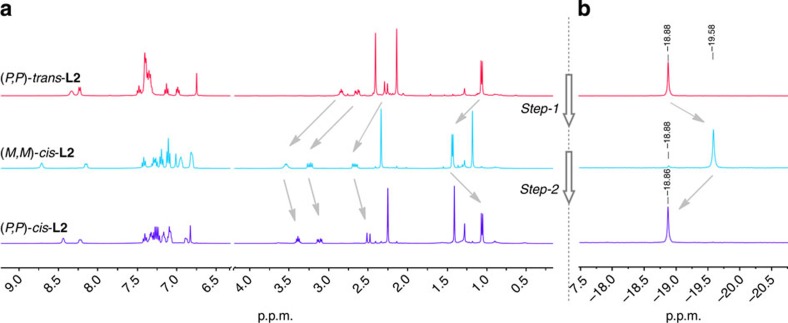
NMR spectra of switching step-1 and step-2. (**a**) Partial ^1^H NMR (CD_2_Cl_2_) spectra of compounds (*P*,*P*)-*trans*-**L2**, (*M*,*M*)-*cis*-**L2** and (*P*,*P*)-*cis*-**L2** on irradiation of (*P*,*P*)-*trans*-**L2** at 312 nm in CH_2_Cl_2_ and after thermal helix inversion ((*R*,*R*)-(*M*,*M*)-*cis*-**L2→**(*R*,*R*)-(*P*,*P*)-*cis*-**L2**) on heating at 45 °C for 14 h. (**b**) The corresponding ^31^P NMR (CD_2_Cl_2_) spectra of compounds (*P*,*P*)-*trans*-**L2**, (*M*,*M*)-*cis*-**L2** and (*P*,*P*)-*cis*-**L2**, respectively (H_3_PO_4_ was used as an external standard).

With the switchable bisphosphines in hand, their photochemical and thermal isomerization properties were investigated. Surprisingly, photoisomerization of **L1** at *λ*_max_=312 nm (−15 °C, THF) showed that only 7% of (*P*,*P*)-*trans*-**L1** was converted to (*M*,*M*)-*cis*-**L1** after reaching the photostationary state (PSS), based on ^1^H NMR analysis. On the contrary, ligand **L2** with a reversed amide bond linker, was functioning as an effective molecular motor, which underwent the expected four-stage rotary cycle uncompromised (Fig. 3). The 360° unidirectional rotation cycle of **L2** includes two photoisomerization steps and two thermal isomerization steps as characterized by ultraviolet–visible, circular dichroism (CD), ^1^H NMR and ^31^P NMR spectroscopy. The first photoisomerization step was performed by irradiation of enantiomerically pure (*P*,*P*)-*trans*-**L2** with ultraviolet light (*λ*_max_=312 nm, −15 °C) in THF, which resulted in a significant decrease in the intensity of the absorption band at 280 nm and the appearance of a new absorption band at 350 nm (Fig. 4a). This red shift is typical for the formation of the (*M*,*M*)-*cis*-**L2** isomer^7^,^8^,^21^,^32^. ^1^H NMR and ^31^P NMR studies in CD_2_Cl_2_ also confirmed this structure as is evident from the downfield shift of all the aliphatic ring protons in ^1^H NMR and the shift of the phosphorus absorption in ^31^P NMR from −18.88 to −19.58 p.p.m. (Fig. 5). After reaching the PSS, a ratio of 93% (*M*,*M*)-*cis*-**L2** and 7% (*P,P*)*-trans*-**L2** was established by ^1^H NMR and ^31^P NMR. The observed changes in CD spectrum also confirm the *P*,*P* to *M*,*M* helix inversion during this step (new band at 350 nm, Fig. 4e). The excellent (*M*,*M*)-*cis*-**L2** to (*P*,*P*)-*trans*-**L2** ratio in the PSS state is highly beneficial in applications of catalytic asymmetric reactions. On heating of (*M*,*M*)-*cis*-**L2** in THF at 65 °C for 1 h, the thermal helix inversion from (*M*,*M*)-*cis*-**L2** to (*P*,*P*)-*cis*-**L2** resulted in a blue shift in the ultraviolet–visible spectrum (Fig. 4b). The thermal helix inversion was also confirmed by the upfield shifts of all the signals of the aliphatic ring protons in the ^1^H NMR spectrum, the ^31^P shift from −19.58 to −18.86 p.p.m. and the CD spectral changes (Figs 4f and 5). The helix inversion step-2 resulted in a quantitative transformation from (*M*,*M*)-*cis*-**L2** to (*P*,*P*)-*cis*-**L2** based on ^1^H NMR and ^31^P NMR measurements. A kinetic study for the thermal isomerization step-2 provided the standard Gibbs energy of activation (Δ^‡^*G*°=100.2 kJ mol^−1^) and half-life of (*M*,*M*)-*cis*-**L2** (*t*_½_=362 h at 0 °C). The long half-life is essential for further application of this isomer in enantioselective catalysis studies. The subsequent photoisomerization step-3, which was carried out at −60 °C on irradiation at 312 nm, and the following thermal isomerization step-4 at 0 °C regenerated the initial (*P*,*P*)-*trans*-**L2** and completed the full four-step rotary cycle. These two final steps were also monitored with ultraviolet–vis, CD and ^1^H NMR spectroscopy (Fig. 4c,d,g,h). (M,M)-*trans*-**L2** is highly unstable (Δ^‡^*G*°=83.4 kJ mol^−1^, *t*_½_=11 min at 0 °C) under ambient conditions and as a consequence not suitable for further catalytic studies (see Supplementary Figs 1 and 2).

### Switchable asymmetric catalysis

Having confirmed the four-step unidirectional rotary cycle of bisphosphine **L2**, we investigated its performance as a chiral switchable ligand in asymmetric catalysis. Based on the stability of three out of four isomers only, (*P*,*P*)-*trans*-**L2**, (*M*,*M*)-*cis*-**L2** and (*P*,*P*)-*cis*-**L2** were used in the palladium-catalysed desymmetrization of *meso*-cyclopent-2-en-1,4-diol bis(carbamate) **8,** a well-established model reaction to determine the enantiodiscrimination abilities of chiral ligands^28^,^33^. Initially, the desymmetrization reaction of biscarbamate **8** was carried out with 2.5 mol% of Pd_2_(dba)_3_ and 7.5 mol% of (*R*,*R*)-(*P*,*P*)-*cis*-L**2** in THF. To our delight, the reaction proceeds with high selectivity affording chiral oxazolidinone **9** in 89% yield with an enantiomeric ratio (e.r.) of 11/89. Encouraged by this initial result, several parameters were examined including temperature and base to further enhance the selectivity (see the Supplementary Table 1)^34^. The reaction in the presence of 2.0 equiv. TEA (pKa=10.8) as the base led to a slight increase in the enantioselectivity. Since the base involves deprotonation of nonionized urethane (pKa≈3.7; ref. 35) in the cyclization reaction, screening of a number of other bases revealed that *N*,*N*-diisopropylethylamine (pKa=10.8) was the best choice increasing the e.r. significantly to 6/94.

**Figure 6:**
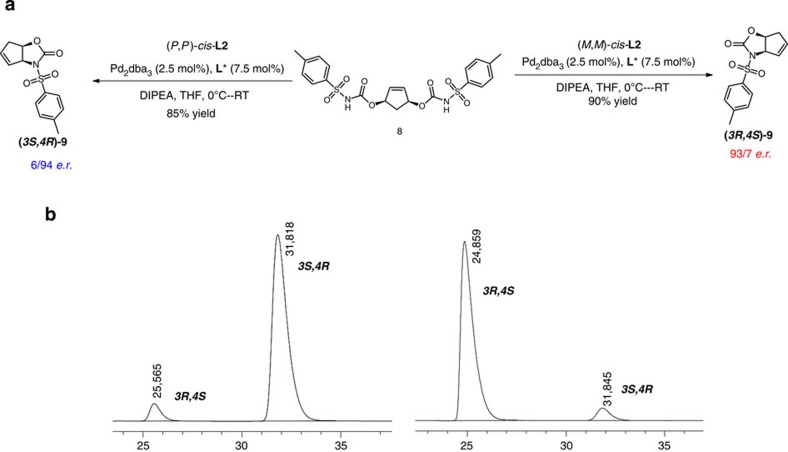
Switchable asymmetric catalysis. (**a**) Stereodivergent synthesis of (*3S,4R*)-and (*3R,4S*)-oxazolidinone **9** by switching the chirality of ligand *cis*-**L2**. (**b**) HPLC trace for **9** from (*R*,*R*)-(*P*,*P*)-*cis*-**L2** and (*R*,*R*)-(*M*,*M*)-*cis*-**L2**. Enantiomeric excess was determined by HPLC using a Chiracel OD-H column. The absolute configuration of the product **9** was confirmed by comparing the sign of optical rotation with literature reported data^34^.

Having established the optimized conditions, the catalytic performance of this tunable chiral ligand was investigated with different isomers (Fig. 6). It should be noted that the *in situ* switching experiment in the presence of Pd is complicated so far due to the stability of the catalysts generated *in situ* under ultraviolet irradiation and heating, leading to a significant decrease in selectivity. When the (*R*,*R*)-(*P*,*P*)-*trans*-**L2** isomer was used in the Pd-catalysed asymmetric desymmetrization, as expected, nearly racemic product **9** was obtained in 65% yield and e.r. of 53/47 (*3R*,*4S*/*3S*,*4R*). Most probably each phosphine in *trans*-**L2** acts as a monodentate ligand as is supported by precipitation of substantial amounts of oligomeric palladium complexes during the reaction. Much to our delight, after photoisomerization to (*R*,*R*)-(*M*,*M*)-*cis-***L2**, the Pd-complex of **L2** was able to catalyse this reaction with excellent stereocontrol providing (*3R*,*4S*)-product **9** (e.r.=93/7, *3R*,*4S*/*3S*,*4R*). In contrast, after isomerization to (*R*,*R*)-(*P*,*P*)-*cis*-**L2**, the opposite enantiomer (*3S*,*4R*)-**9** is obtained again with excellent enantioselectivities (e.r.=6/94, *3R*,*4S*/*3S*,*4R*). From these results, it is clear that ligand **L2** shows excellent performance as a chiral responsive bidentate phosphine ligand. The combination of photochemical isomerization between *trans* and *cis*-isomers and the thermal helix inversion of (*M*,*M*)-*cis* to (*P*,*P*)-*cis*-isomers enables a single chiral ligand to control the formation of nearly racemic, and each enantiomer of the product of a representative Pd-catalysed transformation. In addition, the order in which the different chiral ligands can be formed (in this case *PP*—*MM*—*PP*) is dictated by the stereogenic centres of the chiral motor core unit.

## Discussion

We have successfully designed a photoresponsive chiral bisphosphine ligand that enables the formation of each enantiomer of a product of a Pd-catalysed reaction with a single chiral ligand. The unique combination of a light-driven molecular motor bridging two phosphine moieties allows switching between multiple stereochemical forms with distinct ligand properties. The photochemical and thermal isomerization during unidirectional rotation around the motor central double bond enable to achieve stepwise control over the helicity of the bisphosphine ligand and spatial distance between these two phosphine groups. The stereoselectivity of the Pd-catalysed desymmetrization reaction can be modulated from nearly racemic for (*P*,*P*)-*trans-*state to up to 93/7 e.r. for (*M*,*M*)-*cis-*state and 6/94 e.r. for (*P*,*P*)*-cis-*state, with enantioselectivities comparable to those of related conventional chiral ligands. These results highlight the proof-of-principle of multistage dynamically tunable and responsive chiral ligands at the molecular level for transition-metal-catalysed asymmetric synthesis. These responsive ligand systems have considerable potential for a wide range of enantioselective transformations based on transition metal bisphosphine catalysts. In addition, these systems hold promise to modulate catalysts activity and switch stereoselectivity with high spatio-temporal control ultimately arriving at a catalyst that can perform multiple functions controlled by light.

## Methods

### Procedure for Pd-catalysed intramolecular cyclization of 8

To a Schlenk tube containing Pd_2_dba_3_ (2.3 mg, 2.5 mol%) and the specific isomer of **L2** (7.0 mg, 7.5 mol%), anhydrous THF (0.4 ml) was added, and the mixture was stirred at 0 °C for 30 min under a nitrogen atmosphere. Then, *N*,*N*-diisopropylethylamine (35 μl, 0.20 mmol) and biscarbamate **8** (49.5 mg, 0.10 mmol) were added under nitrogen. The resulting solution was stirred at 0 °C and allowed to slowly warm to room temperature overnight. The mixture was directly submitted to flash chromatography on silica gel with pentane–EtOAc (3/1) as eluent affording product **9** (65% yield for (*P*,*P*)-*trans***-L2**; 90% yield for (*M*,*M*)-*cis***-L2**; 85% yield for (*P*,*P*)-*cis***-L2**).

### 3-Tosyl-3,3a,6,6a-tetrahydro-2H-cyclopenta[*d*]oxazol-2-one (9)

^1^H NMR (400 MHz, CDCl_3_) δ=7.94 (d, *J*=8.3 Hz, 2H), 7.35 (d, *J*=8.1 Hz, 2H), 6.07–5.96 (m, 2H), 5.28 (d, *J*=7.3 Hz, 1H), 5.10 (t, *J*=6.8 Hz, 1H), 2.81 (dd, *J*=18.7, 6.3 Hz, 1H), 2.69 (d, *J*=18.7 Hz, 1H), 2.44 (s, 3H). ^13^C NMR (101 MHz, CDCl_3_) δ=151.3, 145.5, 135.0, 133.8, 129.7, 128.3, 128.0, 77.3, 77.0, 76.8, 76.7, 66.3, 39.0, 21.7. HRMS (ESI+, *m/z*) calculated for C_13_H_13_NO_4_SNa [M+Na]^+^ 302.0458; found 302.0460. Enantiomeric excess was determined by HPLC (Chiracel OD-H), *n*-heptane/*i-*propanol=85/15, 40 °C, 254 nm, 0.5 ml min^−1^, retention times: *t*_R(*3R*,*4S*)_=25 min, *t*_R(*3S*,*4R*)_ 32 min.

## Author contributions

D.Z. and B.L.F. conceived the project. D.Z. and T.M.N. carried out the experimental work. All the authors contributed to the design of the experiments, the analysis of the data and the writing of the paper.

## Additional information

**How to cite this article:** Zhao, D. *et al.* Dynamic control of chirality in phosphine ligands for enantioselective catalysis. *Nat. Commun.* 6:6652 doi: 10.1038/ncomms7652 (2015).

## Supplementary Material

Supplementary Information Supplementary Figures 1-22, Supplementary Table 1, Supplementary Methods and Supplementary References
