# Supplementary material for: Dynamic control of chirality in phosphine ligands for enantioselective catalysis
Source: Nat Commun. 2015 Mar 25;6:6652. doi: 10.1038/ncomms7652 (PMC4389239; doi:10.1038/ncomms7652)
Supplement: Supplementary Information — Supplementary Figures 1-22, Supplementary Table 1, Supplementary Methods and Supplementary References [file ncomms7652-s1.pdf]

Supplementary Figures

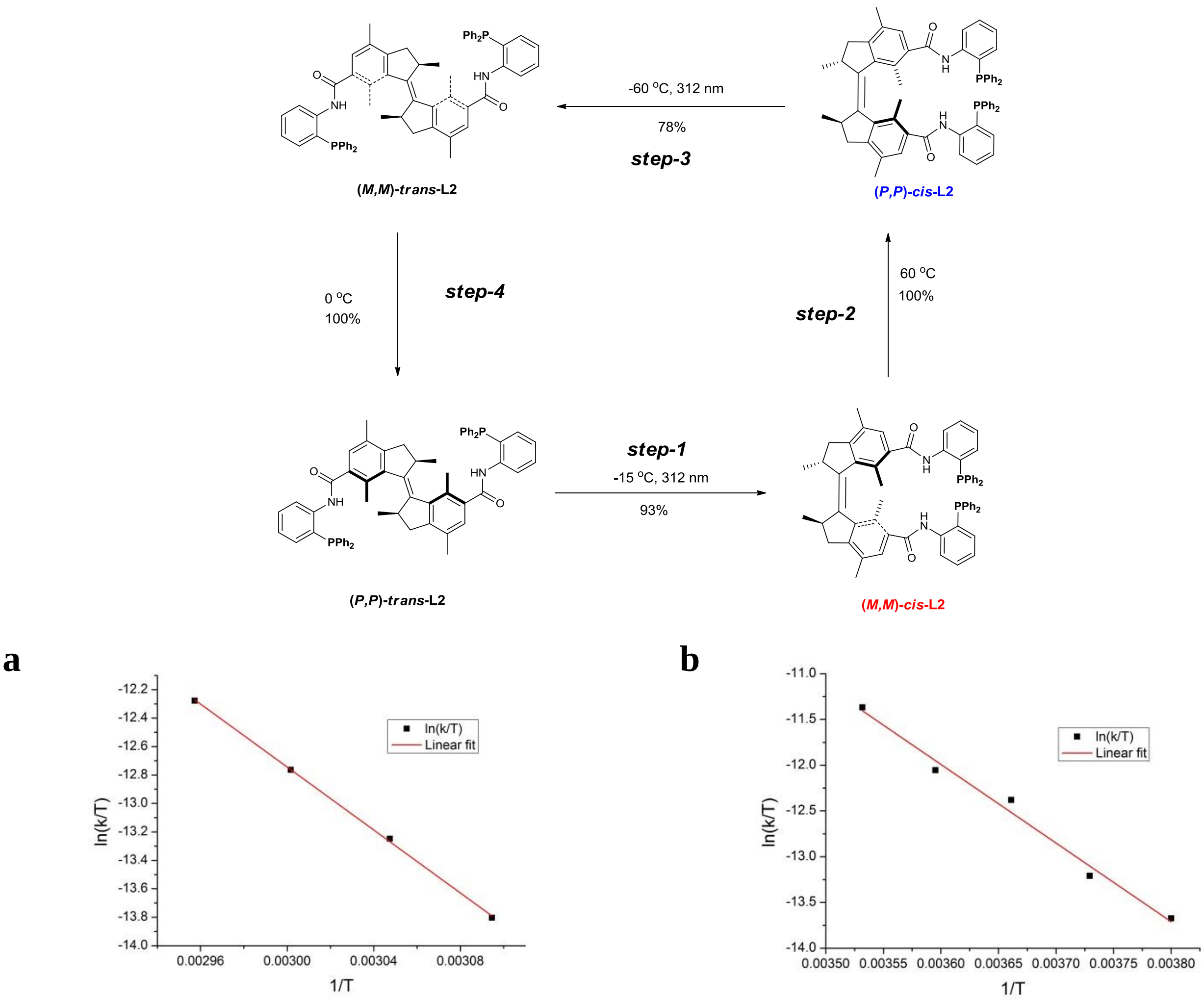

Supplementary Figure 1. Kinetic study for the thermal isomerization steps of **L2.** (**a**) Kinetic study for the thermal isomerizing ***step-2***. The isomerization from (*R*,*R*)-(*M*,*M*)-*cis*-**L2** to (*R*,*R*)-(*P*,*P*)-*cis*-**L2** was followed by UV absorption changes at 350 nm at four different temperatures (50, 55, 60 and 65 °C). The rate constants *k* of the first-order decay at different temperatures were obtained using the equation $A/A^0 = e^{-\kappa t}$. Analysis of these data using Eyring equation ($\Delta^{\ddagger}G = RT$ [ln (k_B/h) - ln (k/T)], provides the standard Gibbs energy of activation ($\Delta^{\ddagger}G°$ = 100.2 kJ/mol). The half-life of (*M*,*M*)-*cis*-**L2** at 0 °C is 362 h and 21 h at 20 °C (obtained by linear fit of ln*k*/T and 1/T). (**b**) Kinetic study for the thermal isomerizing ***step-4***. The isomerization from (*M*,*M*)-*trans*-**L2** to (*P*,*P*)-*trans*-**L2** was followed by UV absorption changes at 350 nm at five different temperatures (-10, -5, 0, 5 and 10 °C). The standard Gibbs energy of activation is $\Delta^{\ddagger}G°$ = 83.4 kJ/mol. The half-life of (*M*,*M*)-*cis*-**L2** is 20 s at 20 °C.

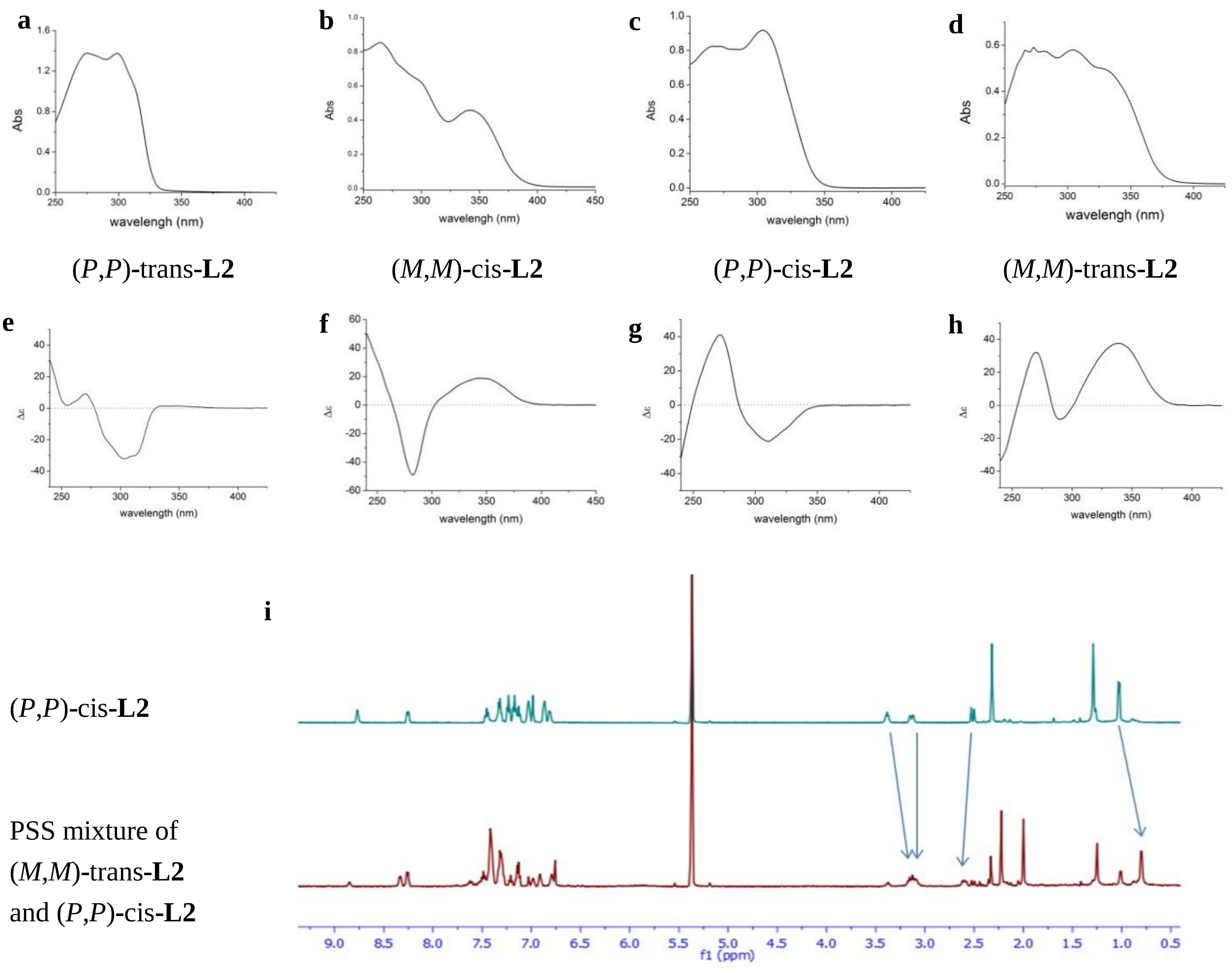

Supplementary Figure 2. Spectral data for photoisomerizations and thermal isomerizations. UV-vis (**a** to **d**) and CD (**e** to **h**) spectra for (*P*,*P*)-trans-**L2**, (*M*,*M*)-cis-**L2**, (*P*,*P*)-cis-**L2**, and (*M*,*M*)-trans-**L2**, respectively (1 $\times 10^{-5}$ M in THF). (**i**) ^{1}H-NMR spectra of PSS mixture of (*M*,*M*)-trans-**L2** and (*P*,*P*)-*cis*-**L2** after irradiation at -60 ^{o}C at 312 nm (2 $\times 10^{-3}$ M in CD_2Cl_2).

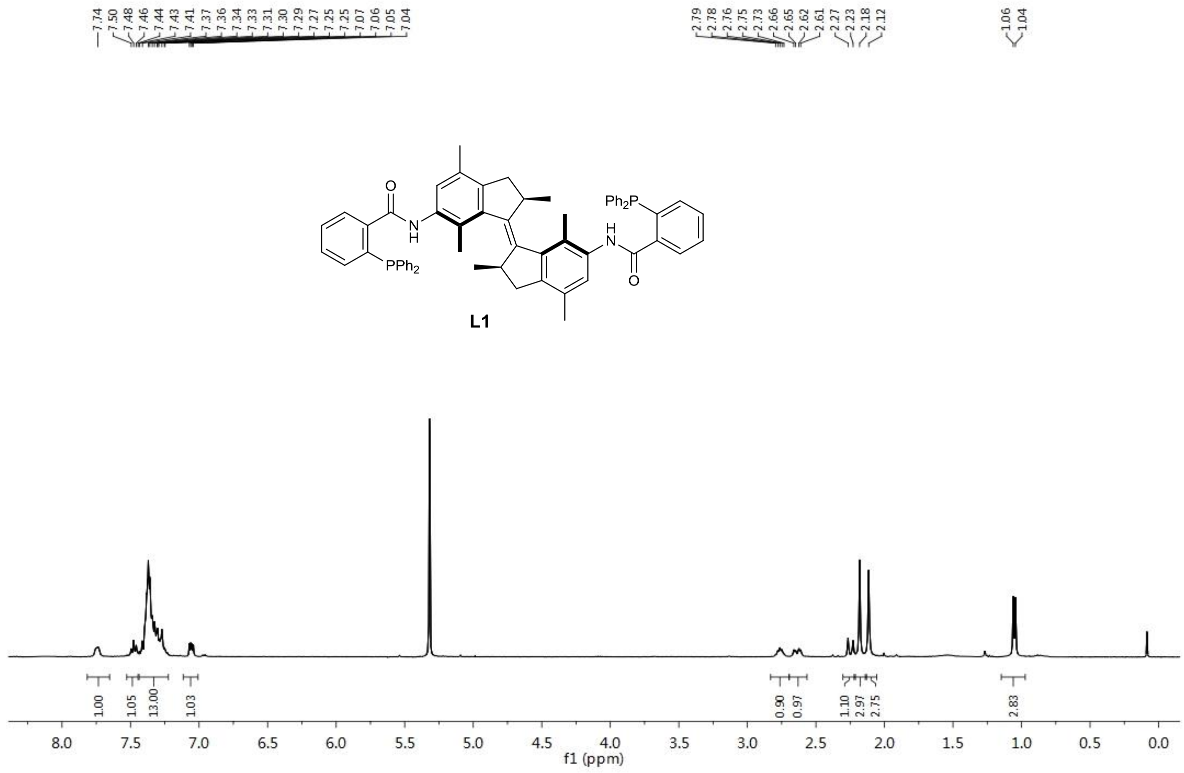

Supplementary Figure 3. ^{1}H NMR spectrum of **L1** (CD_2Cl_2)

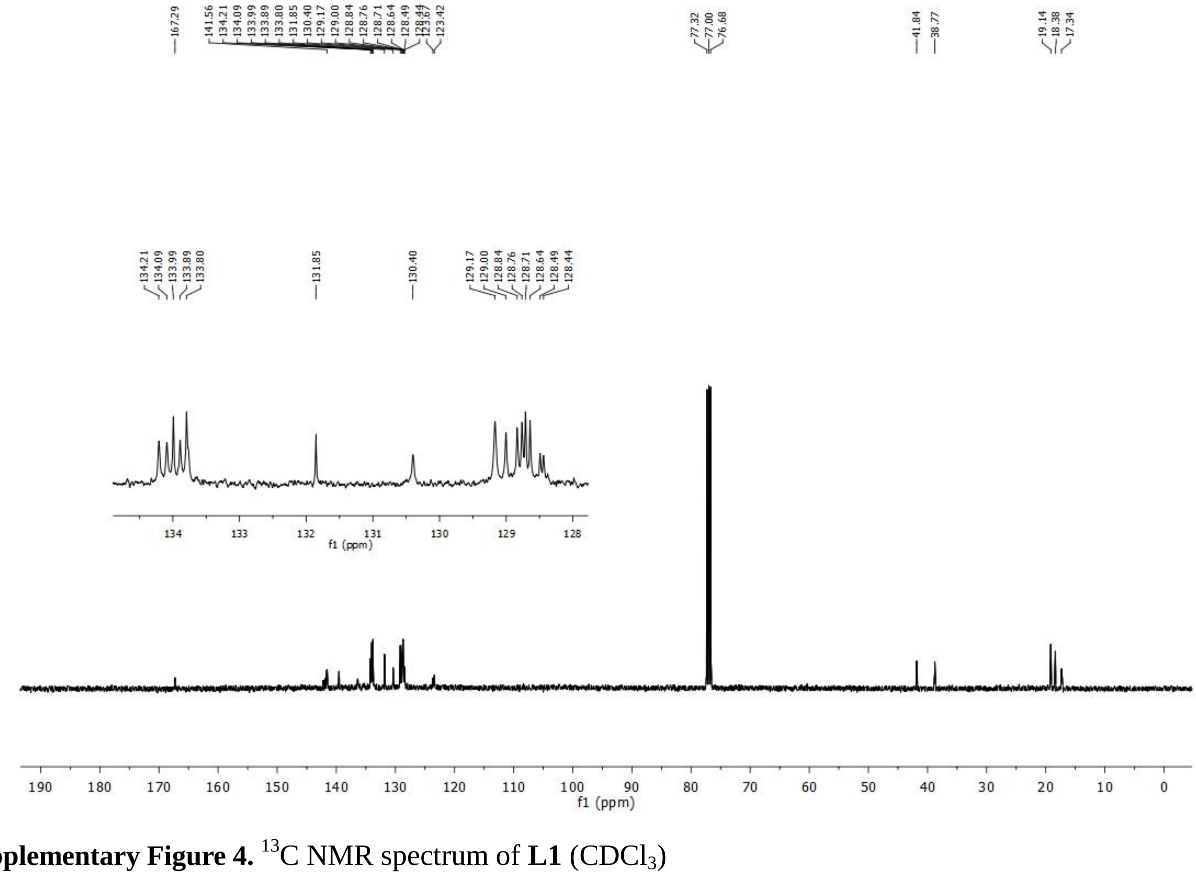

Supplementary Figure 4. ^{13}C NMR spectrum of **L1** ($CDCl_3$)

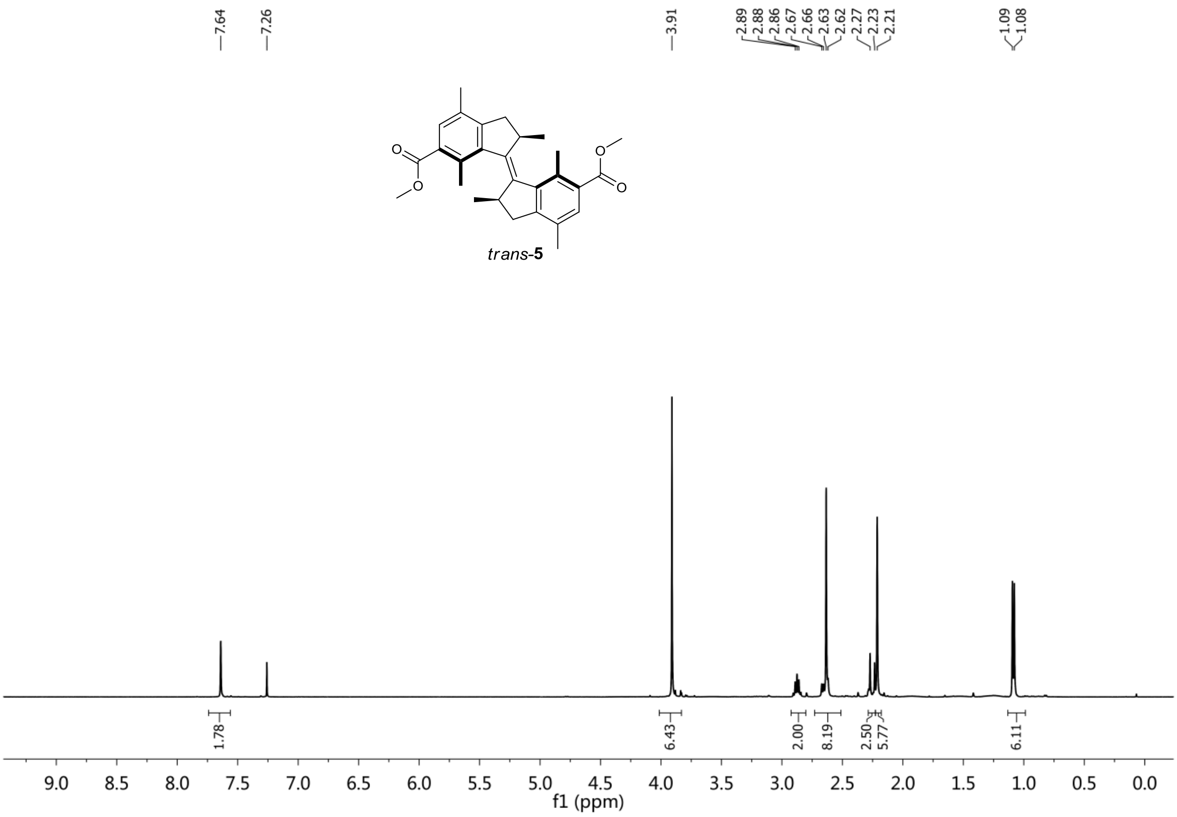

Supplementary Figure 5. ^{1}H NMR spectrum of *trans*-**5** ($CDCl_3$)

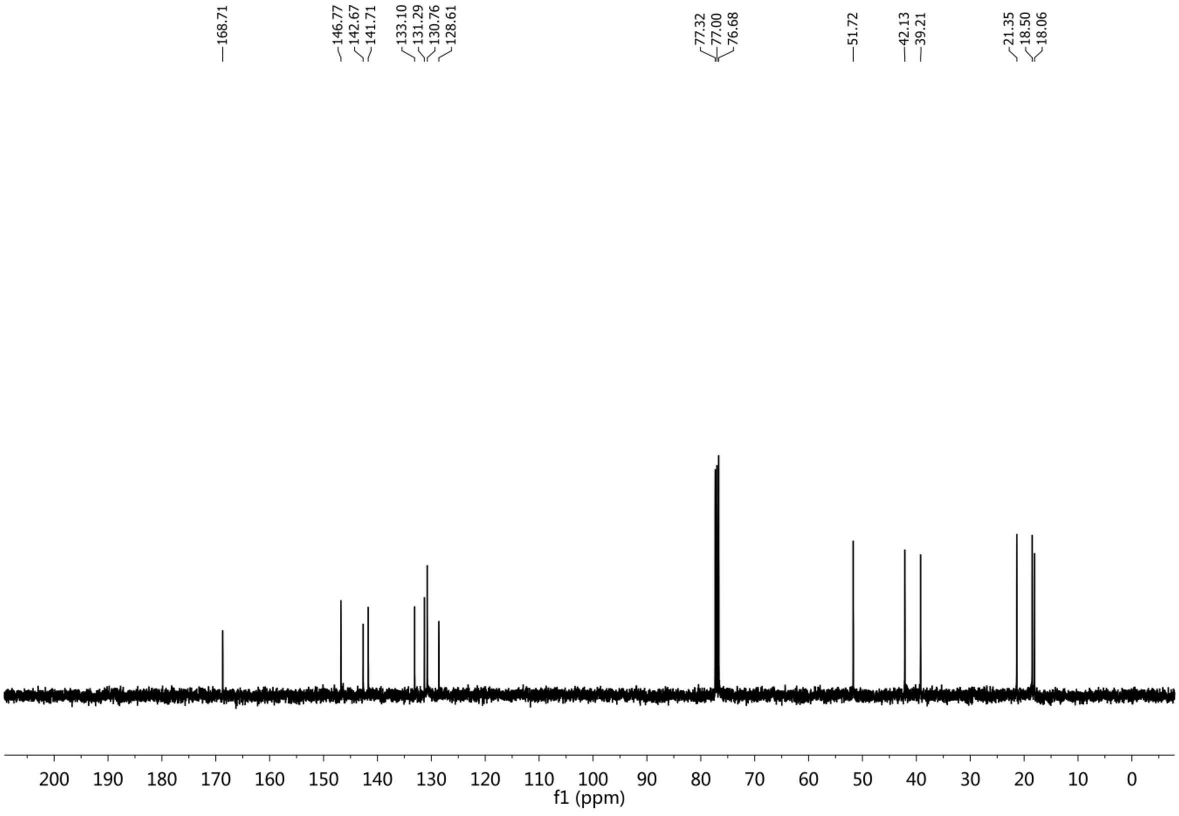

Supplementary Figure 6. ^{13}C NMR spectrum of *trans*-**5** ($CDCl_3$)

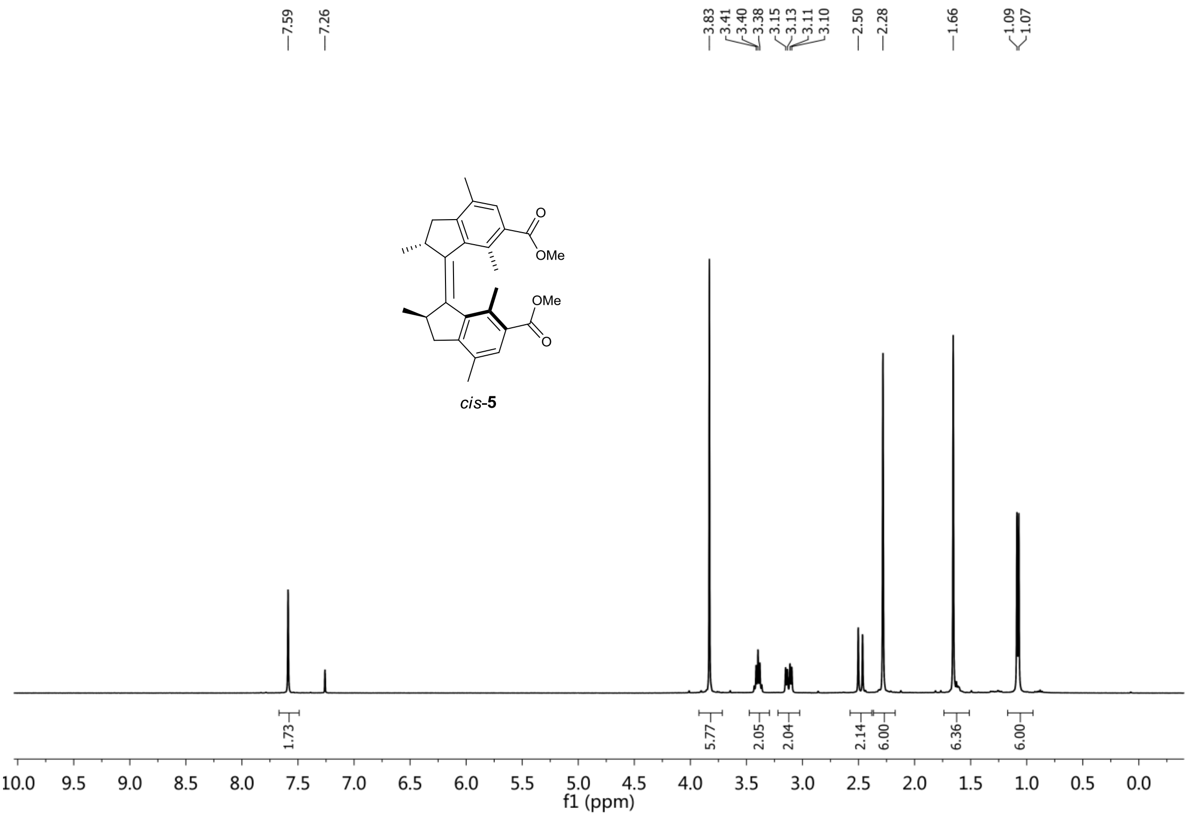

Supplementary Figure 7. ^{1}H NMR spectrum of *cis*-**5** ($CDCl_3$)

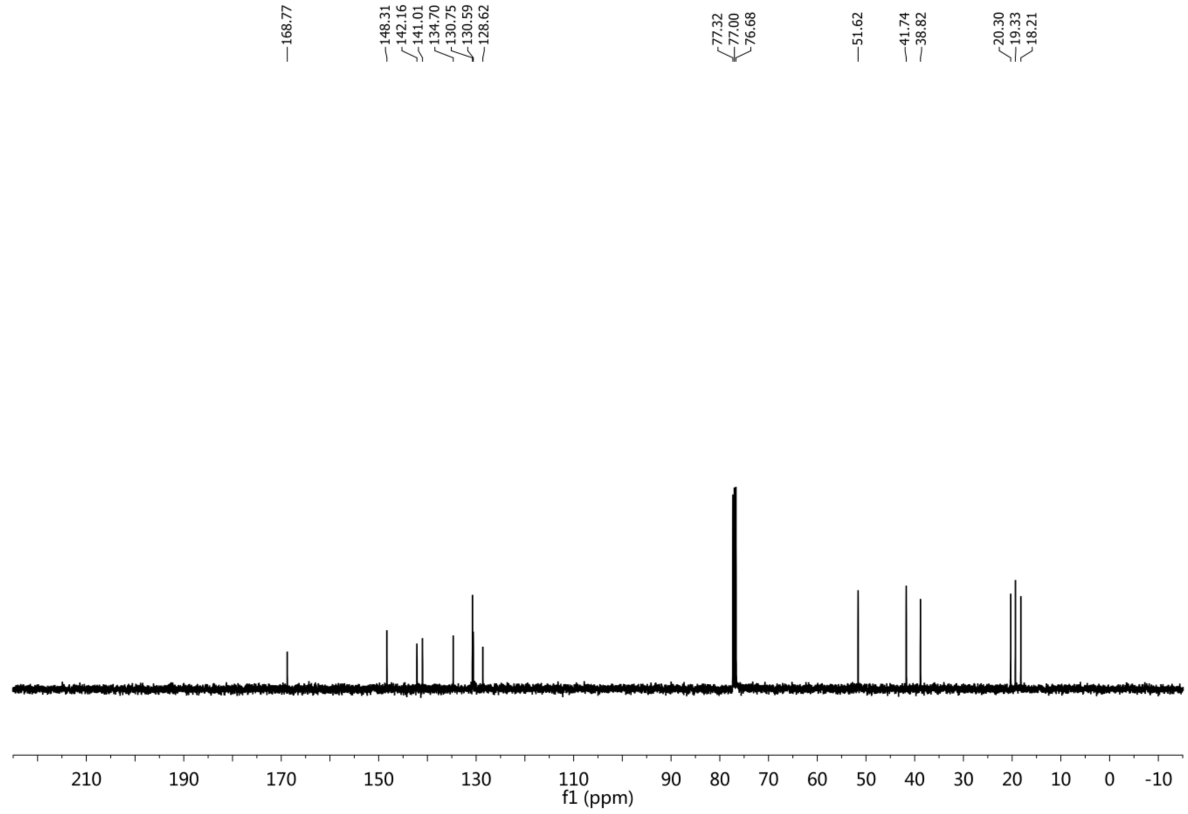

Supplementary Figure 8. ^{13}C NMR spectrum of *cis*-**5** ($CDCl_3$)

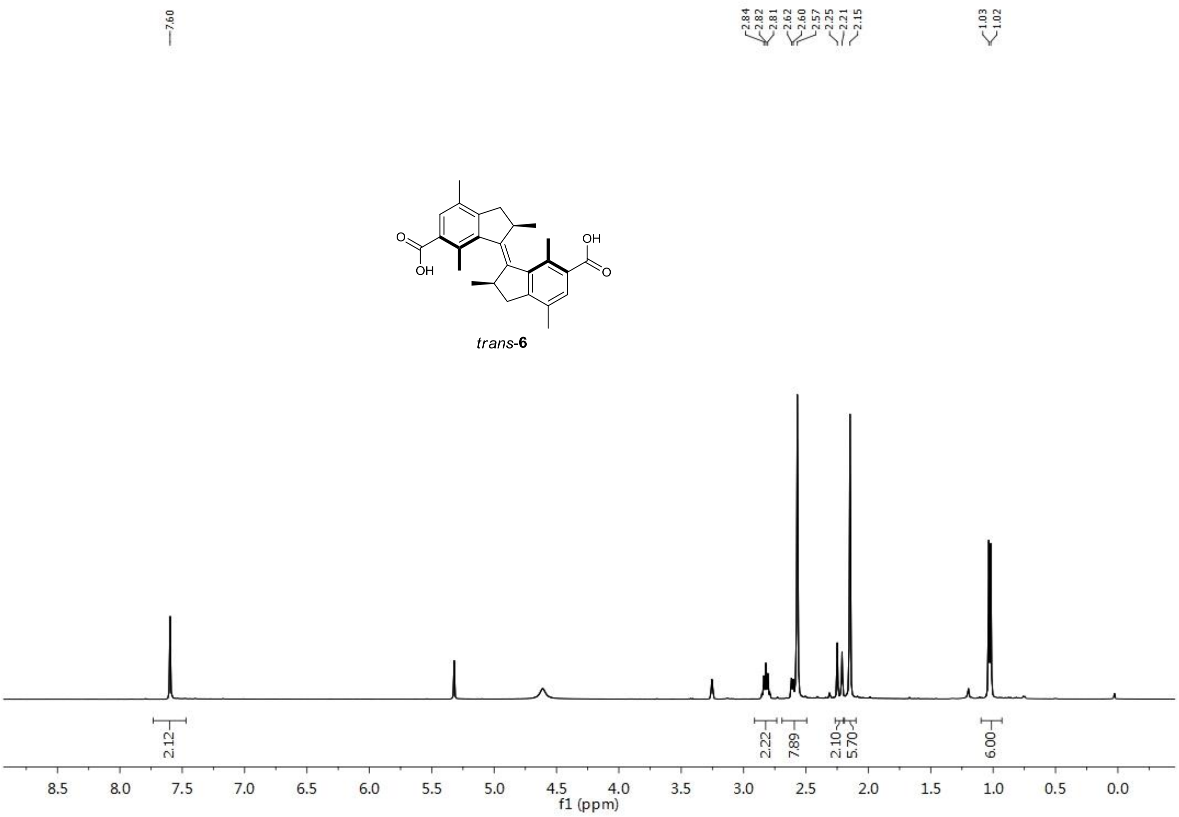

Supplementary Figure 9. ^{1}H NMR spectrum of *trans*-**6** (CD_2Cl_2/ CD_3OD)

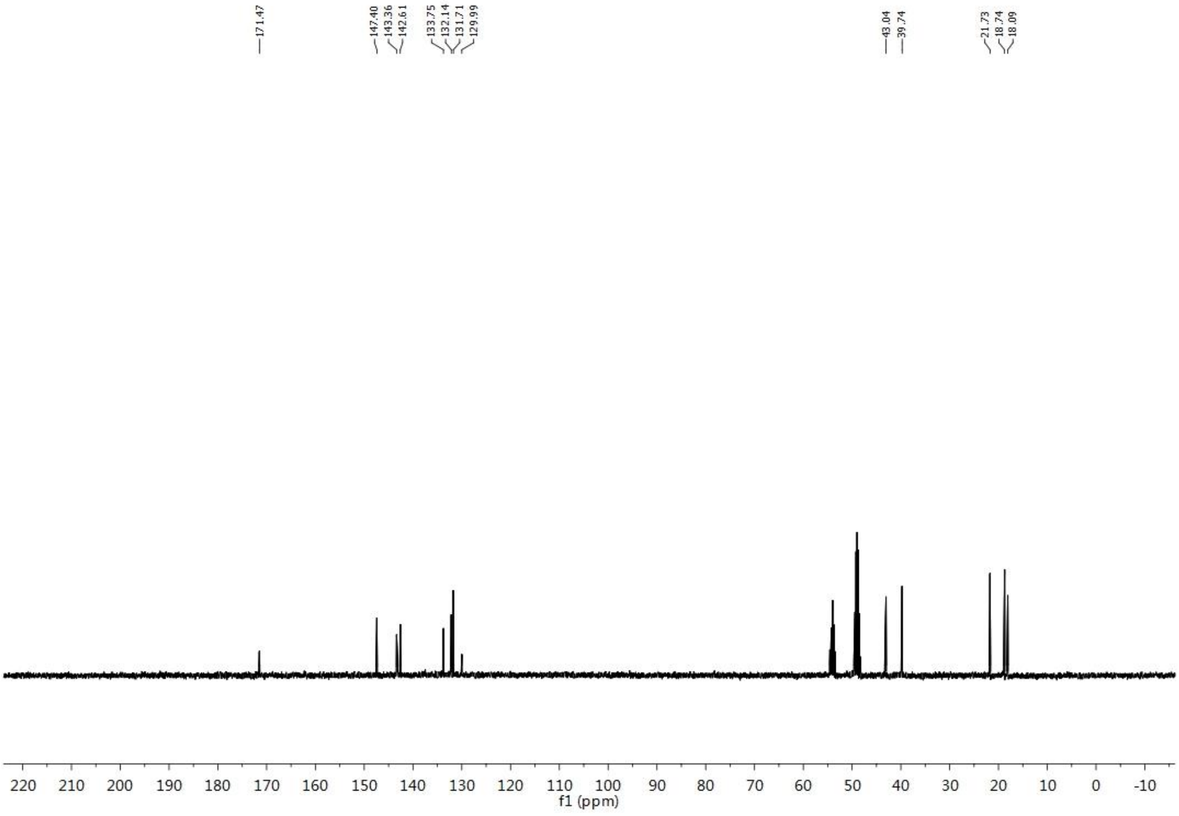

Supplementary Figure 10. ^{13}C NMR spectrum of *trans*-**6** (CD_2Cl_2/ CD_3OD)

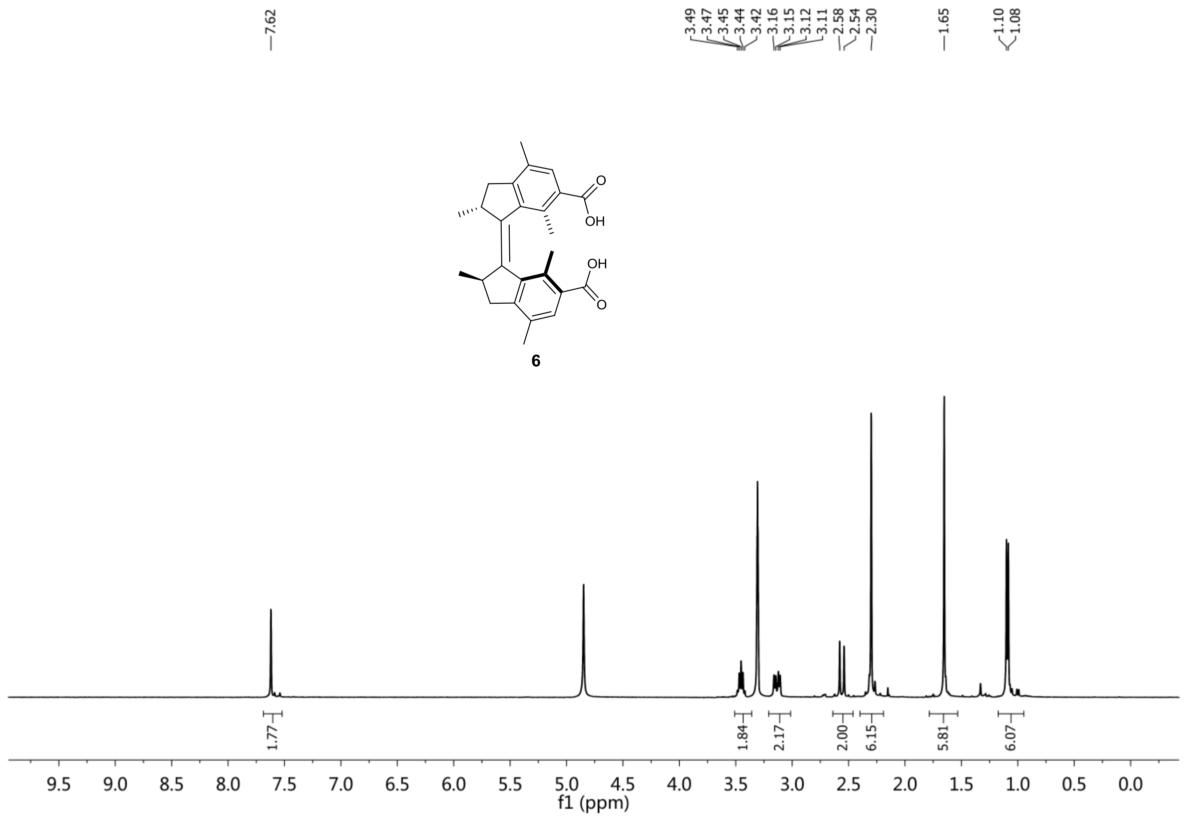

Supplementary Figure 11. ^{1}H NMR spectrum of *cis*-**6** (CD_3OD)

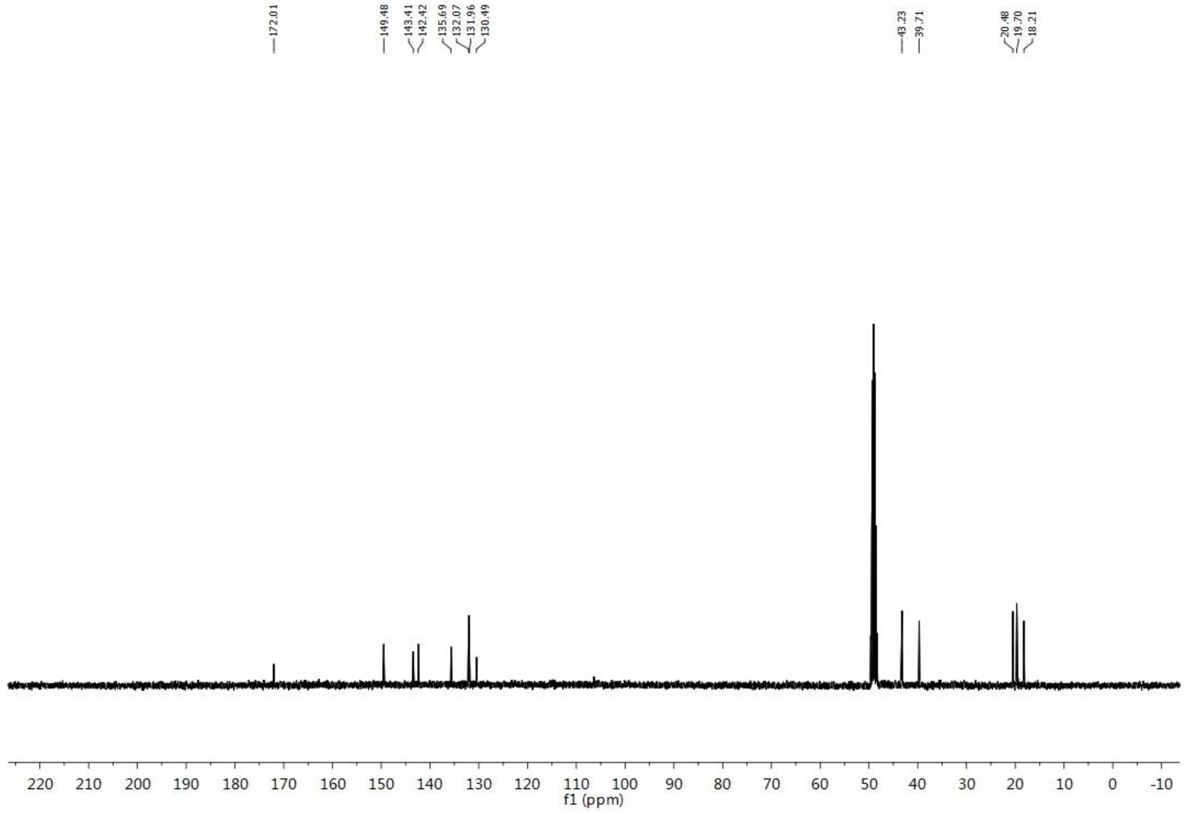

Supplementary Figure 12. ^{13}C NMR spectrum of *cis*-**6** (CD_3OD)

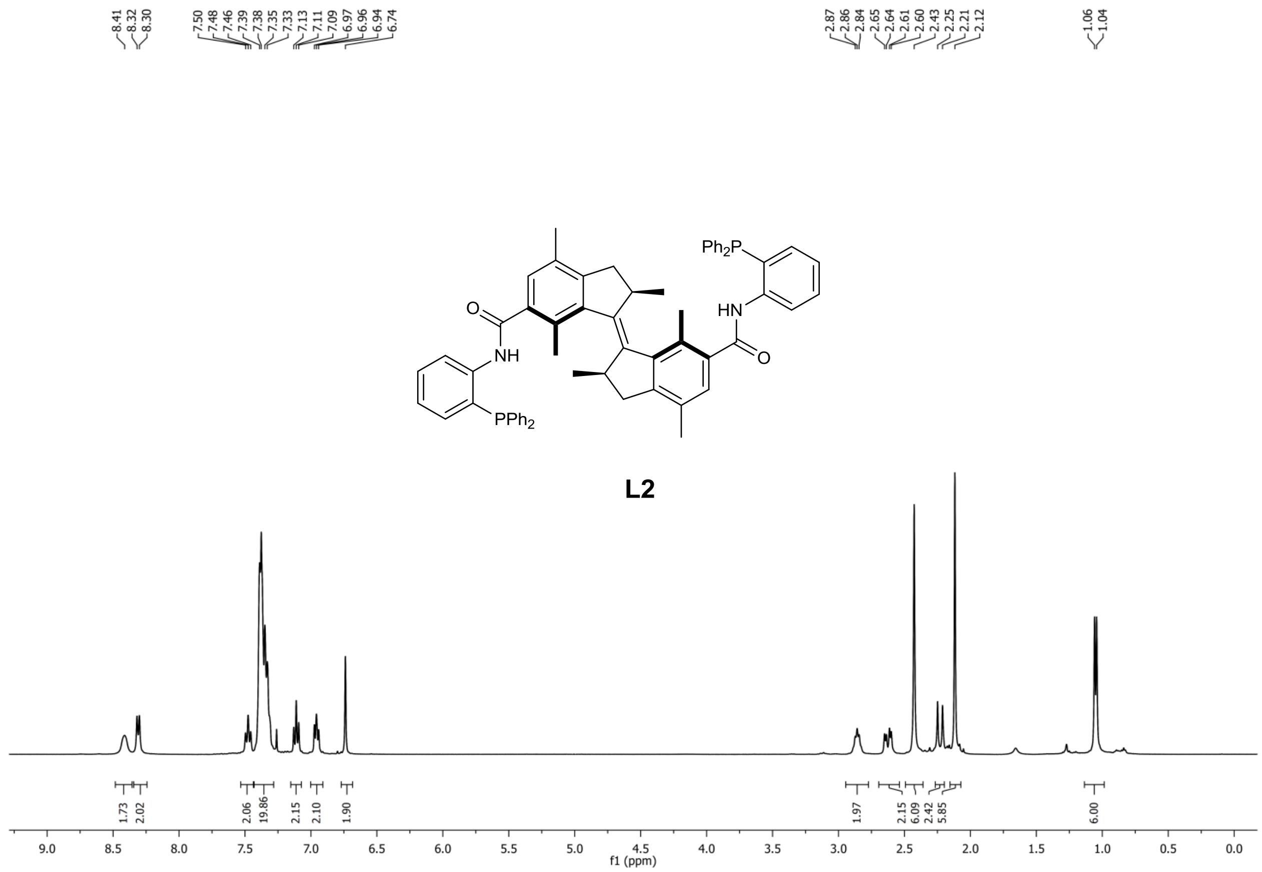

Supplementary Figure 13. ^{1}H NMR spectrum of **L2** ($CDCl_3$)

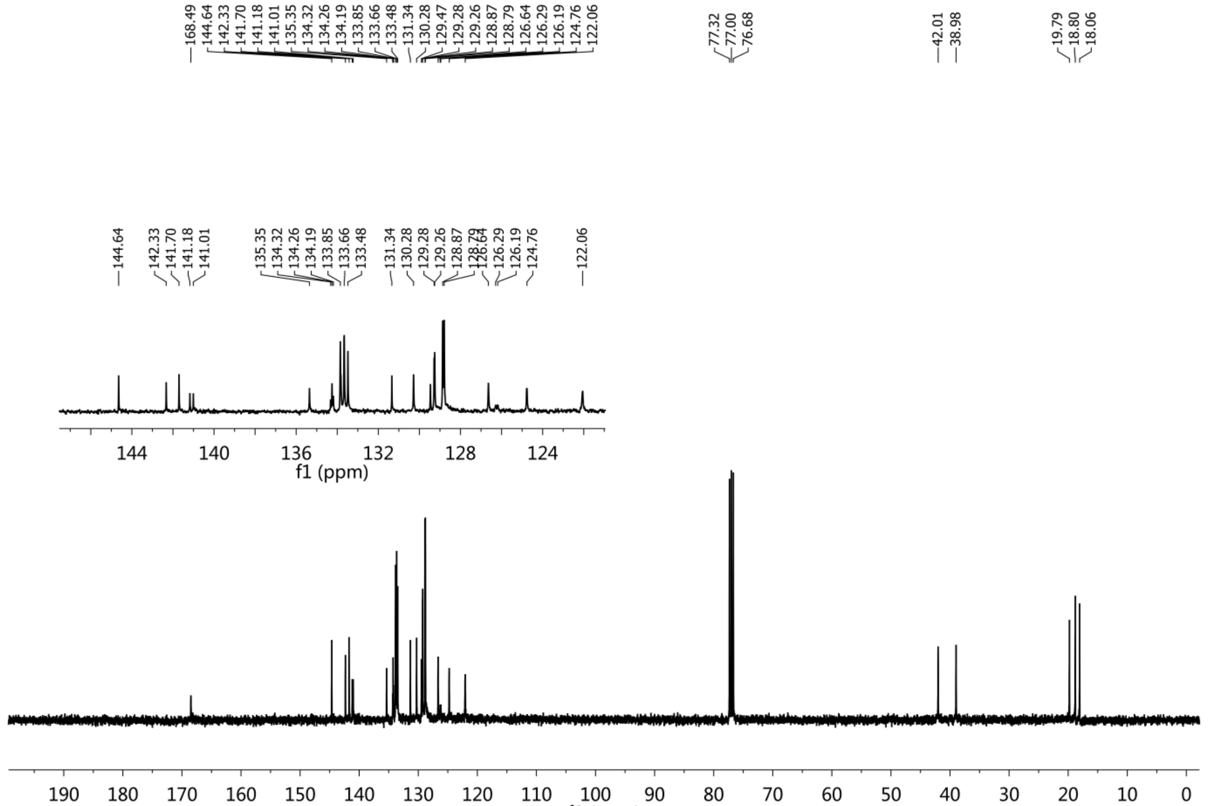

Supplementary Figure 14. ^{13}C NMR spectrum of **L2** ($CDCl_3$)

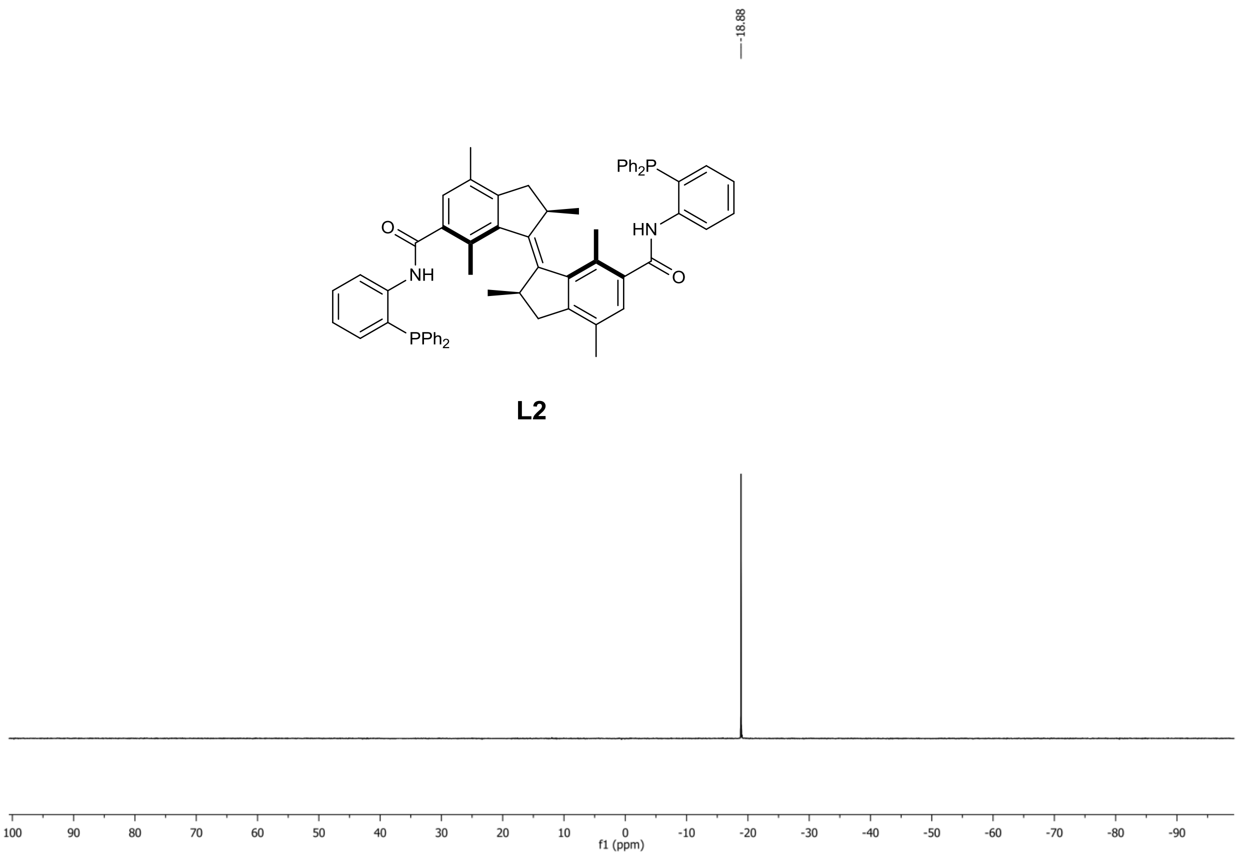

Supplementary Figure 15. ^{31}P NMR spectrum of **L2** (CD_2Cl_2)

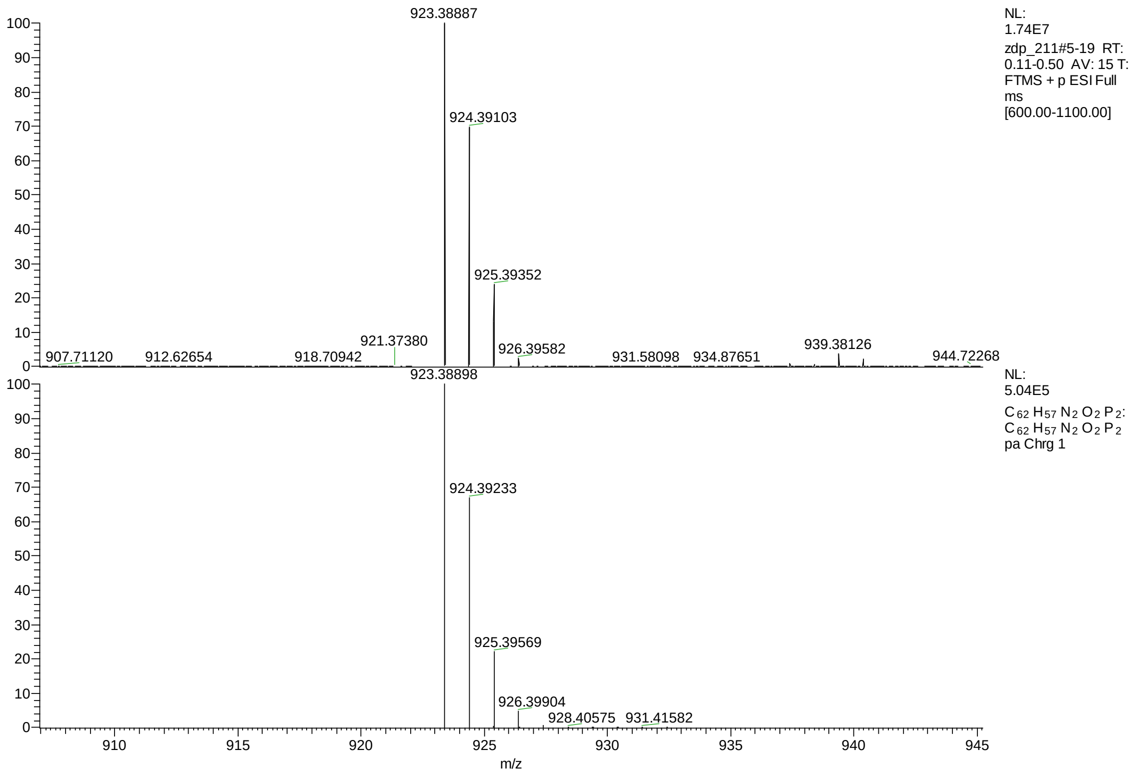

Supplementary Figure 16. HRMS spectrum of **L2**

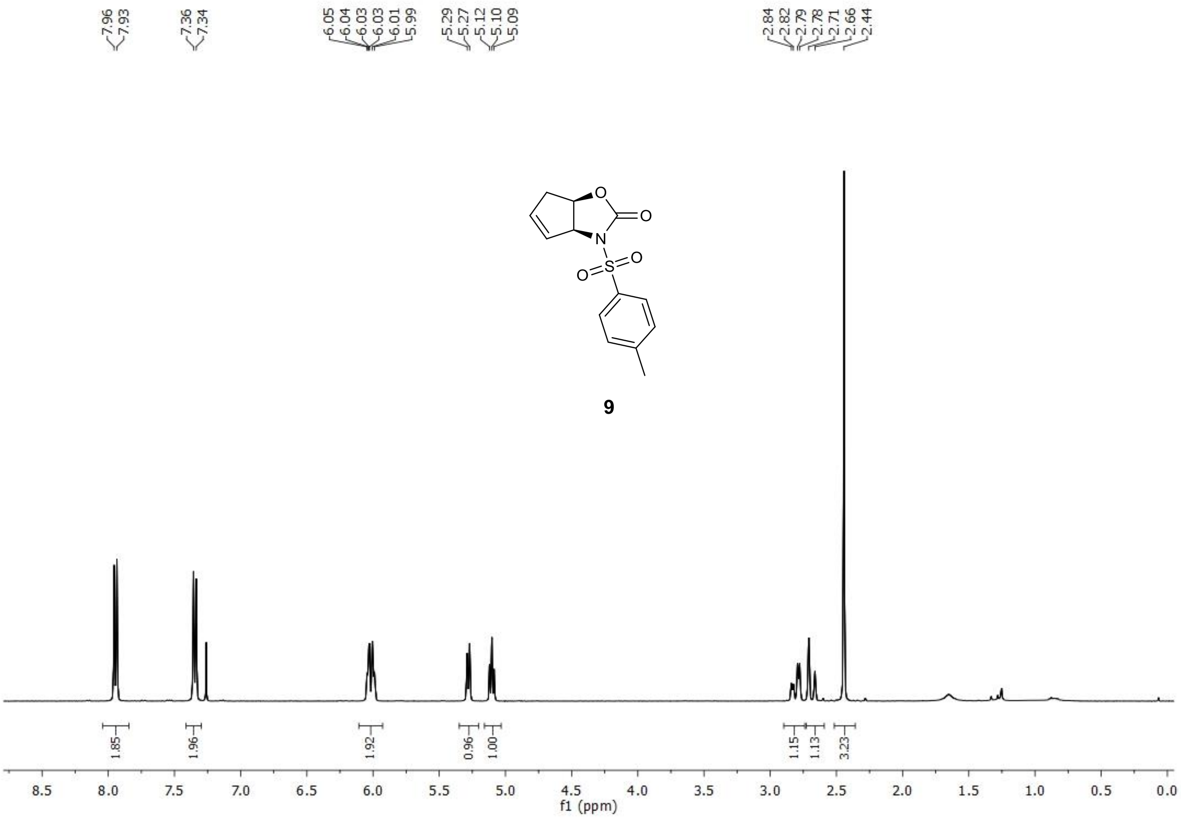

Supplementary Figure 17. ^{1}H NMR spectrum of **9** ($CDCl_3$)

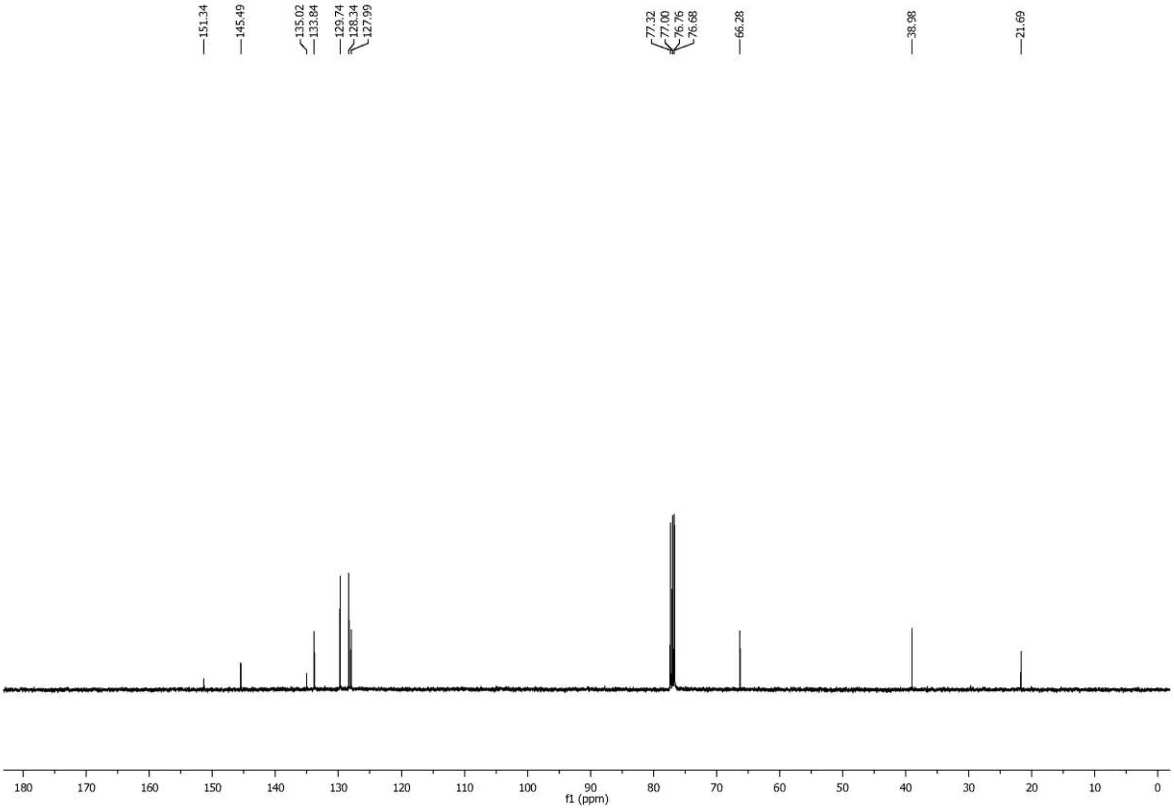

Supplementary Figure 18. ^{13}C NMR spectrum of **9** ($CDCl_3$)

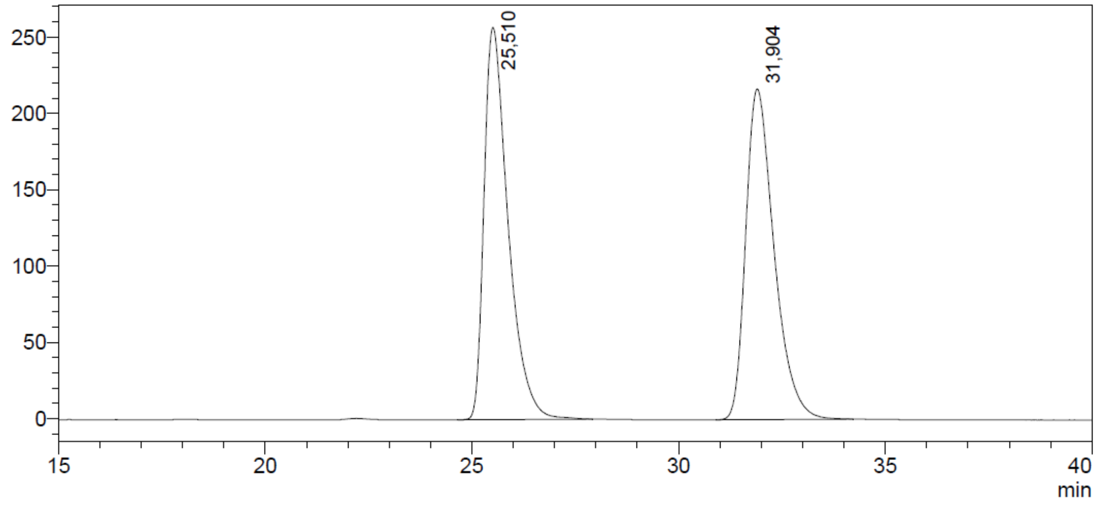

Peak#	Ret. Time	Area	Height	Conc.	Area%
1	25,510	10404418	256911	0,000	50,124
2	31,904	10352914	216558	0,000	49,876
Total		20757333	473469		100,000

Supplementary Figure 19. HPLC trace for *Rac*-**9** (Chiracel OD-H, *n*-heptane/*i*-propanol = 85:15)

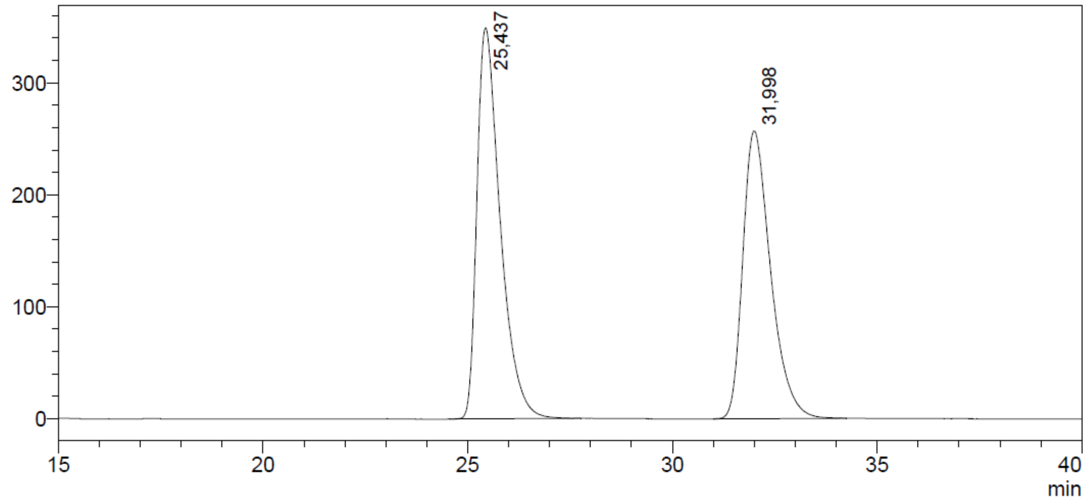

Peak#	Ret. Time	Area	Height	Conc.	Area%
1	25,437	13853209	349482	0,000	53,273
2	31,998	12151147	257109	0,000	46,727
Total		26004356	606591		100,000

Supplementary Figure 20. HPLC trace for **9** from (*R*,*R*)-(*P*,*P*)-*trans*-**L2** (Chiracel OD-H, *n*-heptane/*i*-propanol = 85:15)

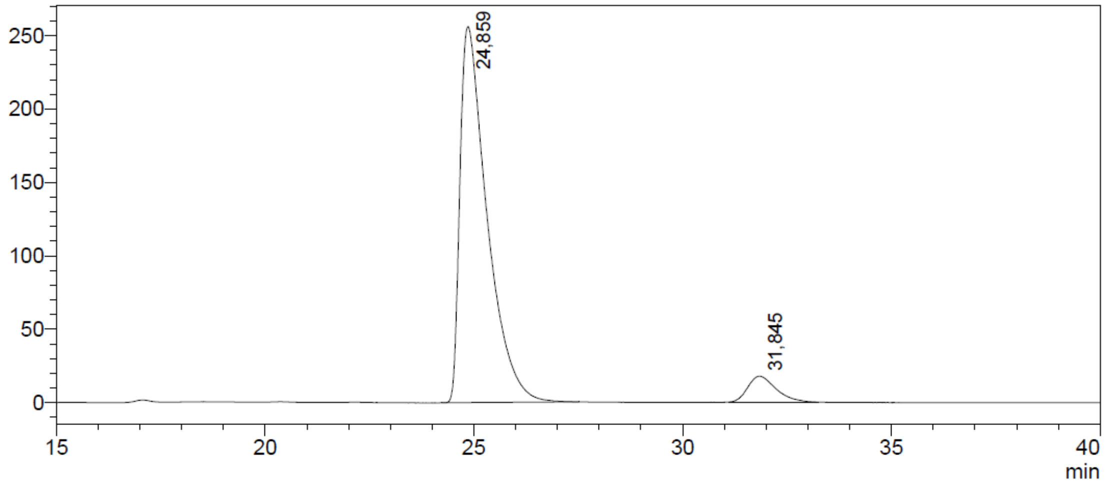

Peak#	Ret. Time	Area	Height	Conc.	Area%
1	24,859	11533777	255990	0,000	93,318
2	31,845	825851	17595	0,000	6,682
Total		12359628	273585		100,000

Supplementary Figure 21. HPLC trace for **9** from (*R*,*R*)-(*M*,*M*)-*cis*-**L2** (Chiracel OD-H, *n*-heptane/*i*-propanol = 85:15)

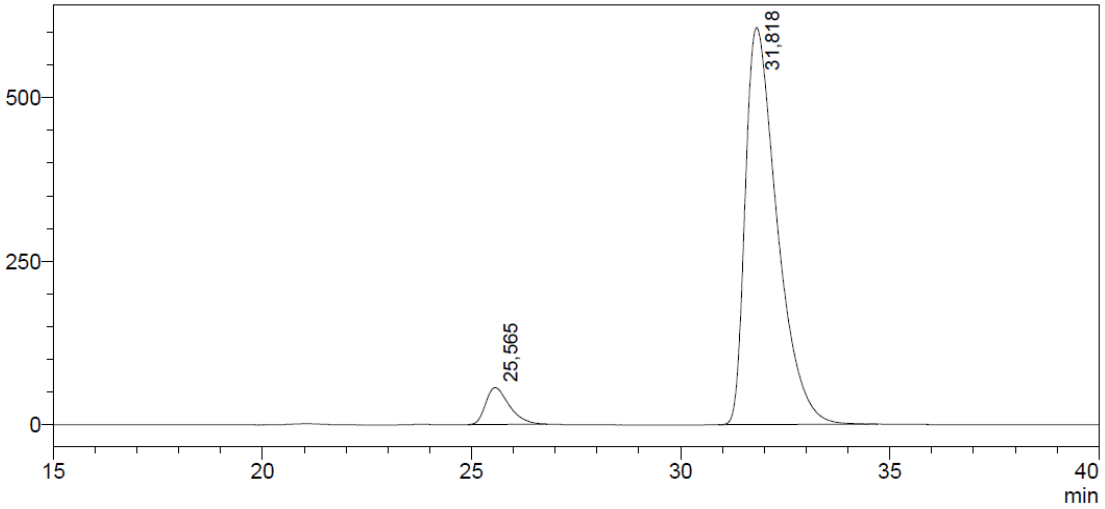

Peak#	Ret. Time	Area	Height	Conc.	Area%
1	25,565	2215744	56416	6,442	6,442
2	31,818	32181765	607741	93,558	93,558
Total		34397509	664157		100,000

Supplementary Figure 22. HPLC trace for **9** from (*R*,*R*)-(*P*,*P*)-*cis*-**L2** (Chiracel OD-H, *n*-heptane/*i*-propanol = 85:15)

Supplementary Table 1. Optimization of the Pd-catalysed asymmetric desymmetrization of *meso*-biscarbamate

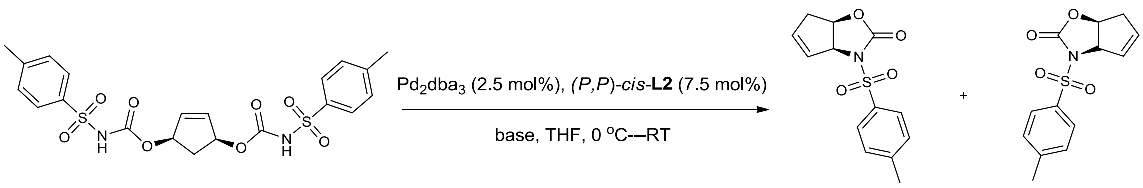

8 **(3S, 4R)-9** **(3R, 4S)-9**

Entry	T (°C)	Base	Yield (%)*	e.r. (*3S,4R*/*3R,4S*) †
1	rt	-	89	89/11
2	rt	Et_3N	81	91/9
3	0 °C	Et_3N	74	92/8
**4**	**rt**	**DIPEA**	**85**	**94/6**
5	rt	$^iBu_3N$	83	89/11

Reactions were performed on 0.1 mmol scale in the presence of $Pd_2(dba)_3$ (2.5 mol%), ligand (7.5 mol%) with 2.0 equivalent of base under the given reaction conditions. *Isolated yields. †Determined by chiral high-performance liquid chromatography (HPLC) on a Chiracel OD-H column.

Supplementary Methods

1. General remarks

Column chromatography was performed on silica gel (Silica-P flash silica gel from Silicycle, size 40-63 μm). TLC was performed on silica gel 60/Kieselguhr F254. Components were visualized by UV and stained with a solution of a mixture of $KMnO_4$ (10 g) and K_2CO_3 (10 g) in H_2O (500 mL). Mass spectra were recorded on a AEI-MS-902 mass spectrometer (EI+) or a LTQ Orbitrap XL (ESI+). ^{1}H, ^{13}C NMR and ^{31}P NMR were recorded on a Varian AMX400 (400 and 100.6 MHz, respectively) or a Varian Unity Plus Varian-500 (500 and 125 MHz, respectively). Chemical shift values for ^{1}H and ^{13}C NMR are reported in ppm with the solvent resonance as the internal standard ($CHCl_3$: δ 7.26 ppm for ^{1}H, δ 77.0 ppm for ^{13}C; acetone: δ 2.05 ppm for ^{1}H, δ 29.8 ppm for ^{13}C; MeOH: δ 3.31 ppm for ^{1}H, δ 49.00 ppm for ^{13}C). Chemical shift values for ^{31}P NMR are reported in ppm using H_3PO_4 as an external standard (δ 0.0 ppm). Data are reported as follows: chemical shifts, multiplicity (s = singlet, d = doublet, t = triplet, q = quartet, br = broad, m = multiplet), coupling constants (Hz), and integration. Optical rotations were measured in $CHCl_3$ on a *Schmidt + Haensch* polarimeter (Polartronic MH8) with a 10 cm cell (*c* given in g/100 mL). Enantiomeric excess values were determined by HPLC analysis using a Shimadzu LC-10AD*VP* HPLC equipped with a Shimadzu SPD-M10A*VP* diode array detector. The analytical supercritical fluid chromatography (SFC) was performed on a Thar Technologies, Inc. (Waters) Investigator II system. Melting points were determined on a Buchi B–545 melting point apparatus. UV measurements were performed on a Jasco V-630 spectrophotometer. All solvents used in reactions were freshly distilled from appropriate drying agents before use. All other reagents were used without further purification. All reactions were performed under anhydrous conditions in a N_2 atmosphere. CD measurements were performed on a Jasco J-815 CD spectrophotometer.

All UV irradiation experiments were carried out using a Spectroline model ENB-280C/FE lamp (8-watt) at 312 nm. Irradiation experiments for UV-vis and CD analysis were performed in dry and degassed THF (1×10^{-5} M) under nitrogen in a quartz cuvette. Irradiation experiments for NMR studies were performed in dry and degassed CD_2Cl_2 (2×10^{-3} M) under nitrogen in a J. Young NMR tube. Thermal isomerization of *step-2* was performed in an oil bath at 40 °C or 60 °C and *step-4* was performed in an ice-water bath.

2. Procedure for the synthesis of (*P*,*P*)-*trans*- L1

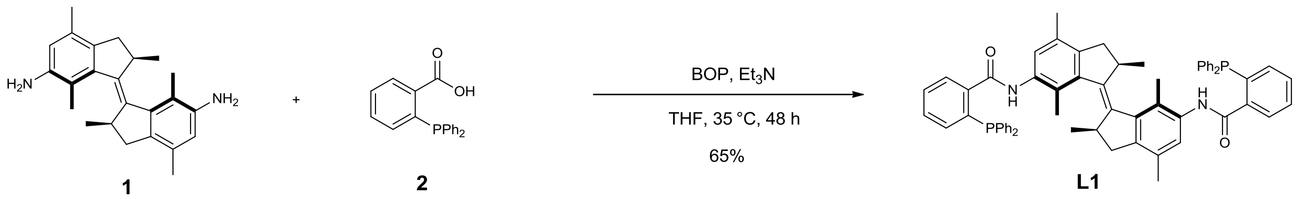

To a mixture of the racemic diamine **1**[1] (173 mg, 0.5 mmol), 2-(diphenylphosphino)benzoic acid **2** (456 mg, 1.49 mmol), and BOP reagent (664 mg, 1.50 mmol) in THF (10 mL) was added dry Et_3N (152 mg, 1.50 mmol) under N_2. The mixture was stirred for 2 d at 35 °C. After cooling to room temperature, it was quenched with brine and extracted with CH_2C1_2 (3 × 50 mL). After drying over Na_2SO_4, the organic solvent was removed *in vacuo* and the residue was purified by flash chromatography on silica gel with EtOAc–pentane (1:10 to 1:4) as eluent to afford the pure product as a white solid in 65% yield. m.p. = 149-151 °C.

^{1}H NMR (400 MHz, CD_2Cl_2) δ = 7.74 (brs, 2H), 7.48 (t, *J* = 7.3 Hz, 2H), 7.44 – 7.22 (m, 26H), 7.06 (dd, *J* = 7.7, 4.0 Hz, 2H), 2.83 – 2.69 (m, 2H), 2.64 (dd, *J* = 14.8, 5.3 Hz, 2H), 2.25 (d, *J* = 14.6 Hz, 2H), 2.18 (s, 6H), 2.12 (s, 6H), 1.05 (d, *J* = 6.4 Hz, 6H).

^{13}C NMR (101 MHz, $CDCl_3$) δ = 167.3, 142.1 (d, *J* = 26.7 Hz), 141.6^{4}, 141.5^{6}, 139.5, 136.4, 135.3 (d, *J* = 19.8 Hz), 134.2 (d, *J* = 12.1 Hz), 134.0, 133.8^{3} (d, *J* = 12.2 Hz), 133.8^{0}, 130.4, 129.2, 129.0, 128.8 (d, *J* = 7.2 Hz), 128.7 (d, *J* = 7.1 Hz),128.5 (d, *J* = 5.2 Hz), 123.7, 123.4, 77.3, 77.0, 76.7, 41.8, 38.8, 19.1, 18.4, 17.3.

^{31}P NMR (81 MHz, $CDCl_3$) δ = -10.81.

HRMS (ESI+, *m/z*) calculated for $C_{62}H_{57}N_2O_2P_2$ $[M + H]^+$ 923.3890; found 923.3902.

3. Procedures for the synthesis of *(R,R)-(P,P)-trans*-L2

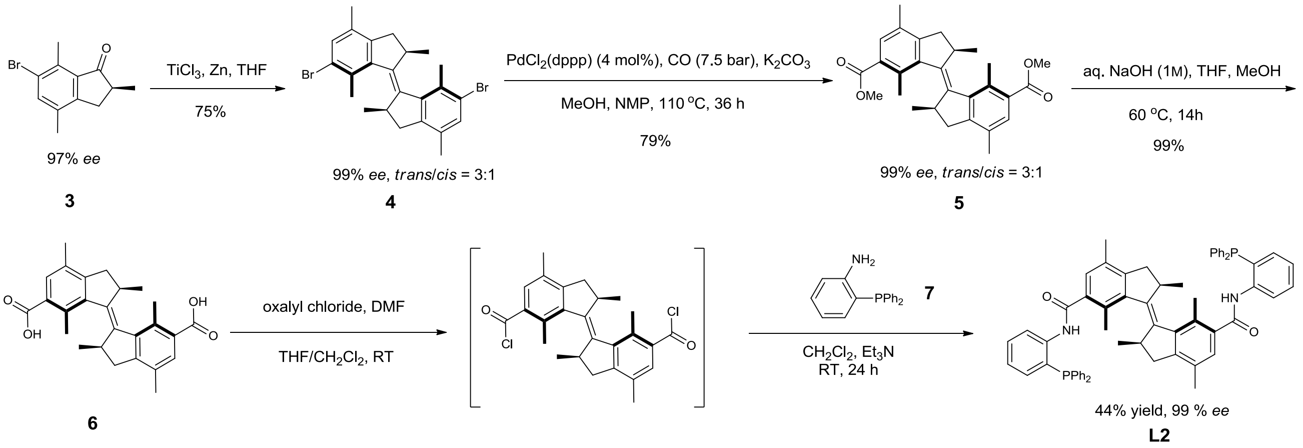

(*R*,*R*)- (*P*,*P*)-*trans*-**4** was prepared according a previous reported procedure.[2]

To a suspension of zinc powder (1.64 g, 25.2 mmol), $TiCl_3$ (1.90 g, 13.0 mmol) in THF (100 mL) was added (*R*)-ketone **3** (1.58 g, 6.2 mmol, 97% ee) at 0 °C. The mixture was heated at reflux for 96 h. After cooling down to room temperature, the reaction mixture was filtered over celite and washed with CH_2Cl_2 (3 × 50 mL). The filtrate was washed with aq. NH_4Cl, which was reextracted with CH_2Cl_2 (3 × 50 mL). The combined organic layers were dried over Na_2SO_4 and concentrated *in vacuo*. The crude product was purified by column chromatography on silica with pentane/ether (50:1) to afford the *E*/*Z*-mixture of **4** (*E/Z* = 75/25) as a white solid. (*P*,*P*)-*trans*-**4**: ^{1}H NMR (400 MHz, $CDCl_3$) δ 7.27 (s, 2H), 2.90 – 2.73 (m, 2H), 2.58 (dd, *J* = 14.6, 5.6 Hz, 2H), 2.46 (s, 6H), 2.22 (d, *J* = 10.8 Hz, 4H), 2.16 (s, 6H), 1.09 (d, *J* = 6.4 Hz, 6H). ^{13}C NMR (101 MHz, $CDCl_3$) δ = 142.6, 142.2, 142.1, 133.2, 131.7, 130.8, 123.5, 42.1, 38.8, 23.0, 18.9, 18.1.

(*P*,*P*)-*cis*-**4**: ^{1}H NMR (400 MHz, $CDCl_3$) δ 7.25 (s, 2H), 3.41 – 3.27 (m, 2H), 3.04 (dd, *J* = 15.1, 6.3 Hz, 2H), 2.41 (d, *J* = 15.2 Hz, 2H), 2.23 (s, 6H), 1.51 (s, 6H), 1.08 (d, *J* = 6.1 Hz, 6H). ^{13}C NMR (101 MHz, $CDCl_3$) δ = 143.3, 141.4, 141.2, 132.6, 132.3, 131.9, 123.5, 41.6, 38.4, 21.3, 20.3, 17.9.

To the reaction flask was added dibromide **4** (1.18 g, 2.50 mmol), $PdCl_2$(dppp) (59.0 mg, 0.10 mmol), K_2CO_3 (759 mg, 5.50 mmol), NMP (10 mL) and anhydrous methanol (2 mL). Once sealed inside the autoclave, the system was purged with argon three times followed by purging with CO. The autoclave was pressurised with CO to 7.5 bar and heated to 110 °C for 36 h. The reaction mixture was stirred under these conditions with additional CO added to maintain the desired pressure. After the indicated time, the reactor was allowed to reach room temperature, and the mixture was diluted with water (10 mL) and extracted with ether (3 × 50 mL). The combined organic phases were

washed with water and brine, dried over Na_2SO_4, concentrated, and the residue was purified by flash chromatography on silica with ethyl acetate/pentane (1:20) to provide (*P*,*P*)-*trans*-**5a** (640 mg) and (*P*,*P*)-*cis*-**5b** (223 mg) in 79% yield.

Dimethyl (2*R*,2'*R*,*E*)-2,2',4,4',7,7'-hexamethyl-2,2',3,3'-tetrahydro-[1,1'-biindenylidene]-6,6'-dicarboxylate (*trans*-5)

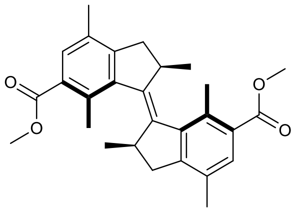

trans-**5**

White solid; m.p. = 62-64 °C; enantiomeric excess was determined by HPLC (Chiracel AD-H), *n*-heptane/*i*-propanol = 97:3, 40 °C, 254 nm, 0.5 mL/min, retention times: t_R (minor) 8.6 min, t_R (major) 11.1 min, ee = 99%; $[\alpha]_D^{20}$ = -114.5 (c 1.0, $CHCl_3$).

^{1}H NMR (400 MHz, $CDCl_3$) δ = 7.64 (s, 2H), 3.91 (s, 6H), 2.92 – 2.80 (m, 2H), 2.73 – 2.51 (m, 8H), 2.25 (d, *J* = 15.1 Hz, 2H), 2.21 (s, 6H), 1.09 (d, *J* = 6.4 Hz, 6H).

^{13}C NMR (101 MHz, $CDCl_3$) δ = 168.7, 146.8, 142.7, 141.7, 133.1, 131.3, 130.8, 128.6, 51.7, 42.1, 39.2, 21.4, 18.5, 18.1.

HRMS (ESI+, *m/z*) calculated for $C_{28}H_{33}O_4$ [M + H]$^+$ 433.2373; found 433.2361.

HPLC trace of (*R*,*R*)-(*P*,*P*)-*trans*-**5a**

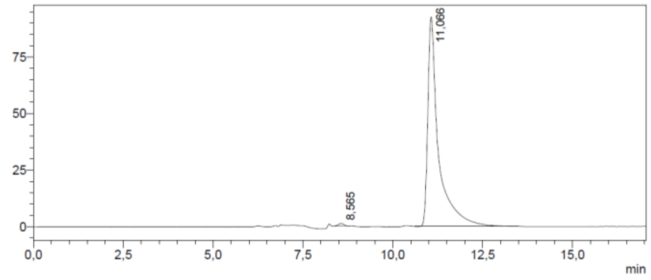

Peak#	Ret. Time	Area	Height	Conc.	Area%
1	8,565	10308	1063	0,000	0,535
2	11,066	1918100	92476	0,000	99,465
Total		1928409	93539		100,000

HPLC trace of *rac*-*trans*-**5a**

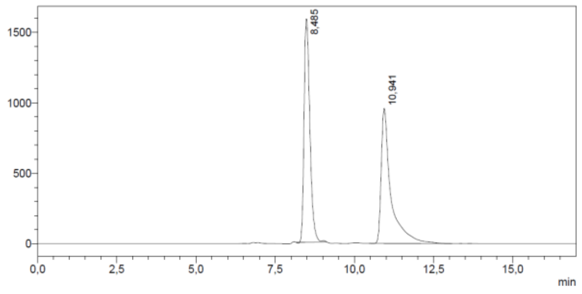

Peak#	Ret. Time	Area	Height	Conc.	Area%
1	8,485	20196283	1584315	0,000	49,680
2	10,941	20456264	957572	0,000	50,320
Total		40652547	2541888		100,000

Dimethyl (2*R*,2'*R*,*Z*)-2,2',4,4',7,7'-hexamethyl-2,2',3,3'-tetrahydro-[1,1'-biindenylidene] -6,6'-dicarboxylate (*cis*-5)

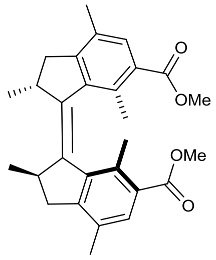

cis-**5**

White solid, m.p. = 170-172 °C; $[\alpha]_D^{20}$ = -225.4 (c 1.0, $CHCl_3$).

^{1}H NMR (400 MHz, $CDCl_3$) δ = 7.59 (s, 2H), 3.83 (s, 6H), 3.47 – 3.29 (m, 2H), 3.12 (dd, *J* = 15.5, 6.4 Hz, 2H), 2.50 (s, 2H), 2.28 (s, 6H), 1.66 (s, 6H), 1.08 (d, *J* = 6.7 Hz, 6H).

^{13}C NMR (101 MHz, $CDCl_3$) δ = 168.8, 148.3, 142.2, 141.0, 134.7, 130.8, 130.6, 128.6, 51.6, 41.7, 38.8, 20.3, 19.3, 18.2.

HRMS (ESI+, *m/z*) calculated for $C_{28}H_{33}O_4$ $[M + H]^+$ 433.2373; found 433.2366.

(2*R*,2'*R*,*E*)-2,2',4,4',7,7'-hexamethyl-2,2',3,3'-tetrahydro-[1,1'-biindenylidene]-6,6'-dicarboxylic acid (6a)

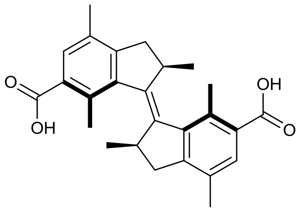

trans-**6**

A solution of *(P,P)-trans*-**5** (440 mg, 1.02 mmol) in MeOH (4 mL), THF (4 mL) and 1 M NaOH (4 mL) was heated to 65 °C for 14 h. After cooling to room temperature, the mixture was acidified to pH 2 by adding aq. HCl (1 M) and extracted with CH_2Cl_2 (3 × 50 mL). The combined organic layer was dried over Na_2SO_4, and filtered, and concentrated to afford *(P,P)-trans*-**6** (408 mg, 99%) as a white solid. m.p. = 269-271 °C. $[\alpha]_D^{20}$ = -123.8 (c 1.0, THF).

^{1}H NMR (400 MHz, CD_3OD/CD_2Cl_2) δ = 7.6 (s, 2H), 2.88 – 2.71 (m, 2H), 2.62 – 2.49 (m, 8H), 2.23 (d, *J* = 15.1 Hz, 2H), 2.15 (s, 6H), 1.01 (d, *J* = 6.5 Hz, 6H).

^{13}C NMR (101 MHz, CD_3OD/CD_2Cl_2) δ = 171.5, 147.4, 143.4, 142.6, 133.8, 132.1, 131.7, 130.0, 43.0, 39.7, 21.7, 18.7, 18.1.

(2*R*,2'*R*,*Z*)-2,2',4,4',7,7'-hexamethyl-2,2',3,3'-tetrahydro-[1,1'-biindenylidene]-6,6'-dicarboxylic acid (6b)

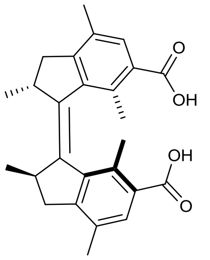

cis-**6**

(*P*,*P*)-*cis*-**6** was obtained using the same procedure as *trans*-**6** in 99% yield as a white solid. m.p. > 300 °C (dec.). $[\alpha]_D^{20}$ = -318.8 (c 1.0, THF).

^{1}H NMR (400 MHz, CD_3OD) δ = 7.62 (s, 2H), 3.57 – 3.37 (m, 2H), 3.13 (dd, *J* = 15.6, 6.4 Hz, 2H), 2.56 (d, *J* = 15.6 Hz, 2H), 2.30 (s, 6H), 1.65 (s, 6H), 1.09 (d, *J* = 6.8 Hz, 6H).

^{13}C NMR (101 MHz, CD_3OD) δ = 172.0, 149.5, 143.4, 142.4, 135.7, 132.1, 132.0, 130.5, 43.2, 39.7, 20.5, 19.7, 18.2.

HRMS (ESI+, *m/z*) calculated for $C_{26}H_{27}O_3$ $[M -OH]^+$ 387.1955; found 387.1950.

(2-Aminophenyl)diphenylphosphane (7)

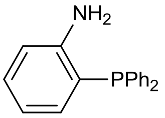

7

(2-Aminophenyl)diphenylphosphane (**7**) was synthesized according to a literature procedure.[3] An oven-dried Schlenk tube was evacuated and refilled with nitrogen three times and then charged with CuI (286 mg, 1.50 mmol, 5 mol%) followed by anhydrous toluene (100 mL), diphenylphosphine (6.2 mL, 36.0 mmol) and *N*,*N*'-dimethylethylenediamine (1.14 mL, 10.5 mmol, 35 mol%). The resulting solution was stirred at room temperature for 10 min. Then 2-iodoaniline (6.6 g, 30 mmol) and Cs_2CO_3 (19.4 g, 60 mmol) were added. The Schlenk tube was sealed and the reaction mixture was stirred at 110 °C for 20 h. The resulting suspension was allowed to reach room temperature, diluted with water (50 mL) and extracted with ethyl acetate (3 × 100 mL). The combined organic phases were dried over Na_2SO_4, concentrated, and the residue was purified by flash chromatography on silica with pentane/ethyl acetate (20:1) to provide the desired product (5.5 g, 66% yield) as a white solid. ^{1}H NMR (400 MHz, $CDCl_3$) δ = 7.41 – 7.29 (m, 10H), 7.18 (t, *J* = 7.6 Hz, 1H), 6.78 (t, *J* = 6.7 Hz, 1H), 6.74 – 6.62 (m, 2H), 4.00 (s, 2H). ^{13}C NMR (101 MHz, $CDCl_3$) δ = 149.7 (d, *J*=19.9 Hz), 135.4 (d, *J*=7.8 Hz), 134.2 (d, *J*=2.6 Hz), 133.6 (d, *J*=19.2 Hz), 130.3, 128.7, 128.5 (d, *J*=7.0 Hz), 119.4 (d, *J*=8.6 Hz), 118.7 (d, *J*=2.1 Hz), 115.4 (d, *J*=2.8 Hz).

^{31}P NMR (162 MHz, $CDCl_3$) δ = -20.37.

(2*R*,2'*R*,*E*)-N^{6},N$^{6'}$-bis(2-(diphenylphosphanyl)phenyl)-2,2',4,4',7,7'-hexamethyl-2,2',3,3'-tetrahydro-[1,1'-biindenylidene]-6,6'-dicarboxamide (L2)

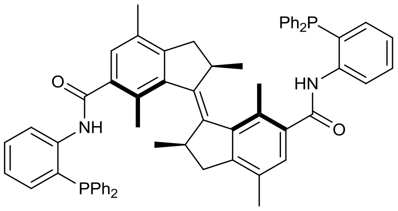

L2

Oxalyl chloride (0.68 mL, 8.0 mmol) was added dropwise to a solution of (*P*,*P*)-*trans*-**6** (404 mg, 1.0 mmol) in anhydrous CH_2Cl_2 (5.0 mL), anhydrous THF (5.0 mL) and one drop of DMF at 0 °C under nitrogen. The reaction mixture was stirred at room temperature for 2 h and subsequently concentrated to afford the crude acid chloride. The resulting acid chloride was dissolved in anhydrous CH_2Cl_2 (10 mL), anhydrous TEA (0.58 mL, 4.00 mmol) and (2-aminophenyl)diphenylphosphane (**7**) (831 mg, 3.00 mmol) was added under nitrogen at 0 °C. The mixture was stirred at room temperature for 24 h. Upon completion, the solvent was evaporated *in vacuo* and the residue was purified by flash chromatography on silica with pentane/ethyl acetate (10:1 to 5:1) to provide the desired product (426 mg, 44% yield). White solid, m.p. = 136-138 °C. Enantiomeric excess determined by chiral SFC analysis, Chiralpak IA (80% CO_2/ 20% MeOH), 230 bar, 40 °C, 3.5 mL/min retention times: t_R (major) 40.2 min, t_R (minor) 50.8 min, ee = 99%. $[\alpha]_D^{20}$ = -14.4 (c 0.5, $CHCl_3$).

^{1}H NMR (400 MHz, $CDCl_3$) δ = 8.41 (brs, 2H), 8.31 (d, *J* = 7.2 Hz, 2H), 7.48 (t, *J* = 7.7 Hz, 2H), 7.43 – 7.28 (m, 20H), 7.11 (t, *J* = 7.5 Hz, 2H), 7.00 – 6.91 (m, 2H), 6.74 (s, 2H), 2.94 – 2.77 (m, 2H), 2.63 (dd, *J* = 14.9, 5.5 Hz, 2H), 2.43 (s, 6H), 2.23 (d, *J* = 14.9 Hz, 2H), 2.12 (s, 6H), 1.05 (d, *J* = 6.4 Hz, 6H).

^{13}C NMR (101 MHz, $CDCl_3$) δ = 168.5, 144.6, 142.3, 141.7, 141.1 (d, *J*= 16.8 Hz), 135.4, 134.3 (d, *J* = 6.9 Hz), 134.2 (d, *J* = 6.9 Hz), 133.8 (d, *J* = 19.2 Hz), 133.5, 131.3, 130.3, 129.5, 129.3 (d, *J* = 2.6 Hz), 128.8 (d, *J* = 7.3 Hz), 126.6, 126.2 (d, *J* = 10.3 Hz), 124.8, 122.1, 42.0, 39.0, 19.8, 18.8, 18.1.

^{31}P NMR (162 MHz, CD_2Cl_2) δ = -18.88.

HRMS (ESI+, *m/z*) calculated for $C_{62}H_{57}N_2O_2P_2$ $[M + H]^+$ 923.3890; found 923.3889.

SFC trace of (*R*,*R*)-(*P*,*P*)-*trans*-**L2**

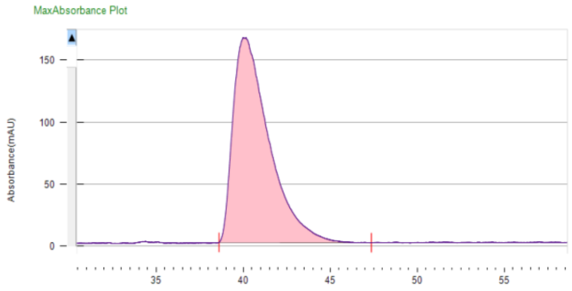

Peak Information

Peak No	% Area	Area	Ret. Time	Height	Cap. Factor
1	100	23825.9726	40.16 min	165.483	40158.6

SFC trace of *rac*-*trans*-**L2**

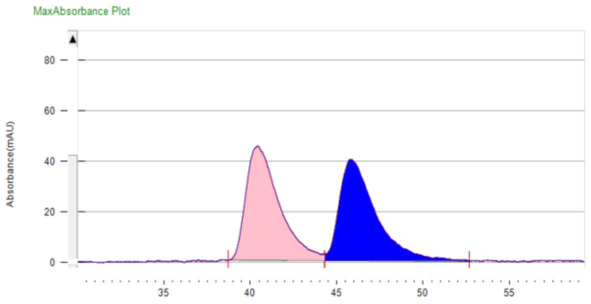

Peak Information

Peak No	% Area	Area	Ret. Time	Height	Cap. Factor
1	50.7776	6047.1577	40.42 min	45.172	40416.9833
2	49.2224	5861.9531	45.8 min	40.0298	45801.4667

4. Procedure for Pd-catalysed intramolecular cyclization of *meso*-biscarbamate 8

To a Schlenk tube containing Pd_2dba_3 (2.3 mg, 2.5 mol%) and the specific isomer of **L2** (7.0 mg, 7.5 mol%) was added anhydrous THF (0.4 mL), and the mixture was stirred at 0 °C for 30 min under a nitrogen atmosphere. Then, DIPEA (35 μL, 0.20 mmol) and biscarbamate **8** (49.5 mg, 0.10 mmol) were added under nitrogen. The resulting solution was stirred at 0 °C and allowed to slowly warm to room temperature overnight. The mixture was directly submitted to flash chromatography on silica gel with pentane–EtOAc (3:1) as eluent affording product **9** (65% yield for (*P*,*P*)-*trans*-**L2**; 90% yield for (*M*,*M*)-*cis*-**L2**; 85% yield for (*P*,*P*)-*cis*-**L2**).

^{1}H NMR (400 MHz, $CDCl_3$) δ = 7.94 (d, *J* = 8.3 Hz, 2H), 7.35 (d, *J* = 8.1 Hz, 2H), 6.07 – 5.96 (m, 2H), 5.28 (d, *J* = 7.3 Hz, 1H), 5.10 (t, *J* = 6.8 Hz, 1H), 2.81 (dd, *J* = 18.7, 6.3 Hz, 1H), 2.69 (d, *J* = 18.7 Hz, 1H), 2.44 (s, 3H).

^{13}C NMR (101 MHz, $CDCl_3$) δ = 151.3, 145.5, 135.0, 133.8, 129.7, 128.3, 128.0, 77.3, 77.0, 76.8, 76.7, 66,3, 39.0, 21.7.

HRMS (ESI+, *m/z*) calculated for $C_{13}H_{13}NO_4SNa$ $[M + Na]^+$ 302.0458; found 302.0460.

Enantiomeric excess was determined by HPLC (Chiracel OD-H), *n*-heptane/*i*-propanol = 85:15, 40 °C, 254 nm, 0.5 mL/min, retention times: $t_{R(3R,4S)}$ 25 min, $t_{R(3S,4R)}$ 32 min.

(*R*,*R*)-(*P*,*P*)-*trans*-**L2** isomer gives nearly racemic product **9** in 65% yield (e.r., *3R*,*4S*/*3S*,*4R* = 53/47); (*R*,*R*)-(*M*,*M*)-*cis*-**L2** gives (*3R*,*4S*)-oxazolidinone **9** (*e.r.*, *3R*,*4S*/*3S*,*4R* = 93/7); (*R*,*R*)-(*P*,*P*)-*cis*-**L2** gives the opposite enantiomer (*3S*,*4R*)-oxazolidinone **9** (*e.r.*, *3R*,*4S*/*3S*,*4R* = 6/94).

Enantiomeric excess was determined by HPLC (Chiracel OD-H), *n*-heptane/*i*-propanol = 85:15, 40 °C, 254 nm, 0.5 mL/min, retention times: $t_{R(3R,4S)}$ 25 min, $t_{R(3S,4R)}$ 32 min. The absolute configuration of the product was confirmed by comparing the sign of optical rotation with literature reported data.[4] Product obtained from (*R*,*R*)-(*P*,*P*)-*cis*-**L2** was determined to be *3S*,*4R* ($[\alpha]_D^{20}$ = +100.2 (c 1.0, CH_2Cl_2); Ref 4, (*3R*,*4S*)-9, $[\alpha]_D^{25}$ = -114.8 (c 2.15, CH_2Cl_2)).

From (*R*,*R*)-(*P*,*P*)-*trans*-**L2** | From (*R*,*R*)-(*M*,*M*)-*cis*-**L2** | From (*R*,*R*)-(*P*,*P*)-*cis*-**L2**

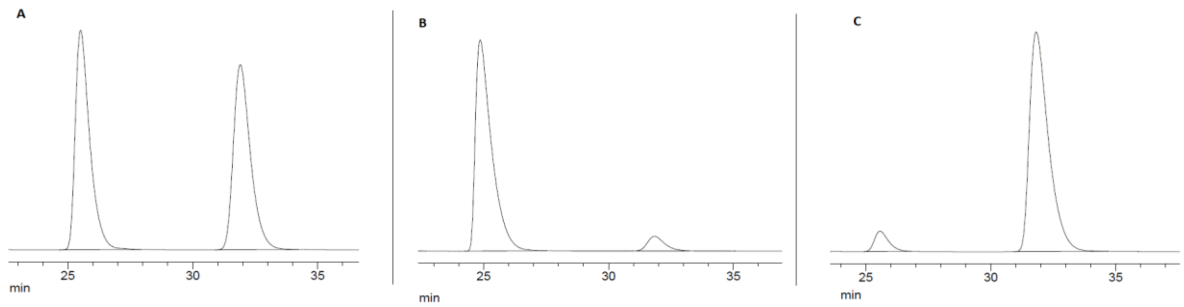

A: HPLC traces of product **9** from (*R*,*R*)-(*P*,*P*)-*trans*-**L2**; **B**: HPLC trace for **9** from (*R*,*R*)-(*M*,*M*)-*cis*-**L2**; C: HPLC trace for **9** from (*R*,*R*)-(*P*,*P*)-*cis*-**L2**.

Supplementary References

1. Vlatković, M. Bernardi, L. Otten, E. & Feringa, B. L. Dual stereocontrol over the Henry reaction using a light- and heat-triggered organocatalyst. *Chem. Commun.* **50**, 7773–7775 (2014).
2. Neubauer, T. M. van Leeuwen, T. Zhao, D. Lubbe, A. S. Kistemaker, J. C. M. & Feringa, B. L. Asymmetric synthesis of first generation molecular motors. *Org. Lett.* **16**, 4220–4223 (2014).
3. Gelman, D. Jiang, L. & Buchwald, S. L. Copper-catalyzed C−P bond construction via direct coupling of secondary phosphines and phosphites with aryl and vinyl halides. *Org. Lett.* **5**, 2315–2318 (2003).
4. Trost, B. M. Patterson, D. E. Enhanced enantioselectivity in the desymmetrization of *meso*-biscarbamates. *J. Org. Chem.* **63**, 1339, (1998).
